# Supplementary material for: Targeting SOX13 inhibits assembly of respiratory chain supercomplexes to overcome ferroptosis resistance in gastric cancer
Source: Nat Commun. 2024 May 20;15:4296. doi: 10.1038/s41467-024-48307-z (PMC11106335; doi:10.1038/s41467-024-48307-z)
Supplement: Supplementary file 1 — Supplementary Information [file 41467_2024_48307_MOESM1_ESM.pdf]

## Supplementary Information

**Supplemental Table S1.** Clinicopathological characterisitcs of 109 GC patients

| Characterisitcs | Number of patients (%) |
|---|---|
| Patients | 109 |
| Gender | |
| Male | 68 (62.39%) |
| Female | 41 (37.61%) |
| Age (years) | 26 to 84, mean 54.78 |
| Tumor size (cm) | 3.2 to 12.5, mean 6.36 |
| Lymph node metastasis (N stage) | |
| N0 | 1 (0.92%) |
| N1 | 29 (26.61%) |
| N2 | 37 (33.94%) |
| N3 | 42 (38.53%) |
| Depth of invasion (T stage) | |
| T1 | 0 (0.00%) |
| T2 | 3 (2.75%) |
| T3 | 18 (16.51%) |
| T4a | 84 (77.06%) |
| T4b | 4 (3.67%) |
| Histology | |
| well and morderately | 54 (49.54%) |
| Poorly and others | 55 (50.46%) |
| Perineural Invasion | |
| Negative | 70 (64.22%) |
| Positive | 39 (35.78%) |
| Lymphovascular invasion | |
| Negative | 66 (60.55%) |
| Positive | 43 (39.45%) |

**Supplementary Table S2.** Clinicopathological characterisitcs of 52 GC patients

| Characterisitcs | Number of patients (%) |
|---|---|
| Patients | 52 |
| Gender | |
| Male | 33 (63.46%) |
| Female | 19 (36.53%) |
| Age (years) | 27 to 73, mean 52.32 |
| Tumor size (cm) | 5.6 to 13.8, mean 8.36 |
| Tumor (pre-NAC,CT) | |
| T4A | 38 (73.07%) |
| T4B | 14 (26.92%) |
| Lymph node (pre-NAC CT) | |
| N0 | 1 (1.92%) |
| N1 | 4 (7.69%) |
| N2 | 41 (78.85%) |
| N3 | 6 (11.54%) |
| TNM stage (pre-NAC CT) | |
| III | 48 (92.31%) |
| IVA | 4 (7.69%) |

**Supplementary Table S3**. Primer sequence used in this study.

| PCR Primers | | Enzyme |
|---|---|---|
| Gene | sequence | |
| β-Actin-F | 5'-GGGAAATCGTGCGTGACATTAAG-3' | |
| β-Actin -R | 5'-TGTGTTGGCGTACAGGTCTTTG-3' | |
| SOX13-F | 5'- CTATGTCCCTCGTAGCCT-3’ | |
| SOX13-R | 5'- AGCACCACCAACTCCC -3’ | |
| SCAF1-F | 5'- CACCAACTAAACTGACCTCCG-3’ | |
| SCAF1-R | 5'- GGGCACACCATCAGCTTTCT-3’ | |
| Cox7a2-F | 5'-CTCGGAGGTAGTTCCGGTTC-3’ | |
| Cox7a2-R | 5'-TCTGCCCAATCTGACGAAGAG-3’ | |
| NADK-F | 5'-CACAATGGGCTGGGTGAGAA-3’ | |
| NADK-R | 5'-TGGACAGGTAGGAGGAGGG-3’ | |
| NADK2-F | 5'-GCTCTACAGTCCGGAAGAACC -3’ | |
| NADK2-R | 5'-GCATCCCAACAACGAGAACG -3’ | |
| SCAF1-F (mice) | 5'- GTTTAGCAGTTTCACGCAGAAG-3’ | |
| SCAF1-R (mice) | 5'-GGCAAATATGATAGGTGGTGCT-3’ | |
| Primers for SCAF1 promoter construct: | | |
| (-1443/+60) SCAF1 sense: | 5’-TATAGATTCCGTAACGGGGCAGGGAG-3’ | Kpnl |
| (-926/+60) SCAF1: | 5’-TATAGATTCCGCTATGGGGTCGGGCTTCTA -3’ | Kpnl |
| (-323/+60) SCAF1 | 5’-TATAGATTCCCTCCTGTTCCGCCACCC-3’ | Kpnl |
| (-103/+60) SCAF1: | 5’- TATAGATTCCACGTCGCCGTGCTCGGTACT -3’ | Kpnl |
| Antisense: | 5’- ATATAGATCTGACTCATCTTCTTCCTCCAT -3’ | Hind III |
| Primers for SCAF1 promoter site-directed mutagenesis: | | |
| binding site 3 mutation sense: | 5’-GGGTCCTTAGGAGAAAATactcCTGGAGATGTGGACTCC-3’ | |
| binding site 3 mutation antisense: | 5’-GGAGTCCACATCTCCAGGagtaTTTTCTCCTAAGGACCC-3’ | |
| binding site 2 mutation sense: | 5’-CTGGAGTGCAATGGgaCGATCTTGGCCCAC -3’ | |
| binding site 2 mutation antisense: | 5’-GTGGGCCAAGATCGTccCATTGCACTCCAG -3’ | |
| binding site 1 mutation sense: | 5’-CCCATGTGTCCCATTacCTTCTGGGAAACCG -3’ | |
| binding site 1 mutation antisense: | 5’-CGGTTTCCCAGAAGGtaATGGGACACATGGG -3’ | |
| Primers used for ChIP in the SCAF1 promoter: | | |
| binding site 3 sense (-1287~-1277): | 5’-GGGTCCTTAGGAGAAAA-3’ | |
| binding site 3 antisense (-1287~-1277): | 5’-CCGGCTCCCTCCTTTA-3’ | |
| binding site 2 sense (-840~-830): | 5’-GGAGTTTCCGTCTTGTAG-3’ | |
| binding site 2 antisense | 5’-CGGGAGGCTGAGGTAG-3’ | |

| | |
|---|---|
| (-840~830): | |
| binding site 1 sense (-153~143): | 5'-GCGACAGAGGCGGTGC-3' |
| binding site 1 antisense (-153~143): | 5'-ACGGCGACGTGAAGGC-3' |
| Vector construction | |
| shRNA-SOX13-1 | 5'- CCGGCCTGCAAACCAGTGGAGTATC CTCGAGGATACTCCACTGGTTTGCAGGTTTTTG -3' |
| shRNA-SOX13-2 | 5'- CCGGACCTCAGCCTTTAGGGCTTATCT CGAGATAAGCCCTAAAGGCTGAGGTTTTTTG -3' |
| siRNA-NADK-1 | 5'- UGAAUGAGGUGGUGAUUGA-3' |
| siRNA-NADK-2 | 5'- CGCCAGCGAUGAAAGCUUU-3' |
| siRNA-NADK-3 | 5'- GAAGACGGCGUGCACAAU-3' |
| siRNA-NADK2-1 | 5'- GCAAUUGCUUCGAUGAUGA-3' |
| siRNA-NADK2-2 | 5'- GAGAAUUGGUAGAGAAAGU-3' |
| siRNA-NADK2-3 | 5'- UGUGAAAGCUGGACGGAUA-3' |
| shRNA-NC | 5'- CAACAAGATGAAGAGCACCAA -3' |
| Lv-SOX13-F | 5'-TATAAAGCTTCTGAGTAGATGTCCATGAGG-3' |
| Lv-SOX13-R | 5'-ATATTCTAGAGGATCAGTCTGTGAGCACCA-3' |
| sg-SCAF1(human) | 5'-GGTGTGGCAAATATGATAGG-3' |
| sg-SCAF1(mice) | 5'-GGTGACGATAGCCCCCGCC-3' |

**Supplementary Table S4.** Information on antibodies.

| Antibody | IHC | WB | CHIP | Specificity | Company (catalog number) |
|---|---|---|---|---|---|
| SOX13 | 1:100 | 1:1000 | 1:50 | Rabbit polyclonal | proteintech (18902-1-AP) |
| COX7A2L/SCAF1 | 1:100 | 1:1000 | / | Rabbit Polyclonal | proteintech (11416-1-AP) |
| NADK | / | 1:1000 | / | Rabbit polyclonal | Abcam (ab233261) |
| NADK2 | / | 1:1000 | / | Rabbit monoclonal | Abcam (ab181028) |
| ACSL4 | / | 1:1000 | / | Rabbit monoclonal | Abcam (ab155282) |
| SLC7A11 | / | 1:1000 | / | Rabbit monoclonal | Abcam (ab175186) |
| GPX4 | / | 1:1000 | / | Rabbit monoclonal | Abcam (ab125066) |
| E-cadherin | / | 1:800 | / | Mouse monoclonal | Abcam (ab1416) |
| Vimentin | / | 1:1000 | / | Rabbit monoclonal | Abcam (ab92547) |
| SDHA | / | 1:1000 | / | Mouse monoclonal | Abcam (ab14715) |
| MTCO1 | / | 1:1000 | / | Mouse monoclonal | Abcam (ab14705) |
| NDUFS2 | / | 1:1000 | / | Mouse monoclonal | Abcam (ab110249) |
| UQCRC2 | / | 1:1000 | / | Mouse monoclonal | Abcam (ab14745) |
| Trim25 | / | 1:1000 | / | Rabbit monoclonal | Abcam (ab167154) |
| FBXO28 | / | 1:800 | | Rabbit polyclonal | Abcam (ab154068) |
| TRAF2 | / | 1:1000 | | Rabbit monoclonal | Abcam (ab126758) |
| β-Actin | / | 1:2000 | / | Rabbit polyclonal | Abcam (ab8227) |

**Supplementary Table S5.** Correlation between SOX13/SCAF1 immunohistochemical scores in 109 GC samples or normal tissue samples. Statistical significance is determined by Pearson correlation analysis.

| | | |
|---|---|---|
| Tumor | r=0.406 | p<0.001 |
| Normal | r=0.162 | p=0.214 |

**Supplementary Table S6.** Correlation between SOX13/SCAF1 coexpression values and Tumor regression Grade. Statistical significance is determined by the nonparametric Mann-Whitney Wilcoxon test.

| SOX13/SCAF1 coexpression value | Tumor Regression Grade | | p |
|---|---|---|---|
| | ≤2 | ≥3 | |
| Low | 22 | 5 | 0.006 |
| High | 11 | 14 | |

Supplementary **Table S7.** Correlation between 4-HNE staining and Tumor regression Grade. Statistical significance is determined by the nonparametric Mann-Whitney Wilcoxon test.

| 4-HNE staining | Tumor Regression Grade | | p |
|---|---|---|---|
| | ⩽2 | ⩾3 | |
| Low | 10 | 16 | 0.003 |
| High | 23 | 3 | |

**Supplementary Table S8.** Correlation between 4-HNE staining and SOX13/SCAF1 IHC scores. Statistical significance is determined by the nonparametric Mann-Whitney Wilcoxon test.

| 4-HNE staining | SOX13/SCAF1 IHC scores | | p |
|---|---|---|---|
| | Low | High | |
| Low | 9 | 17 | 0.025 |
| High | 18 | 8 | |

**Supplementary Table S9.** The 40 compounds with the highest scores.

| Number | Catalog Number | Docking score | Number | Catalog Number | Docking score |
|---|---|---|---|---|---|
| 1 | T1085L | -13.589 | 21 | T1352 | -10.461 |
| 2 | T1663 | -12.578 | 22 | T0278 | -10.369 |
| 3 | T0278 | -12.344 | 23 | T4987 | -10.339 |
| 4 | T5584 | -12.303 | 24 | T4726 | -10.334 |
| 5 | T6654 | -12.100 | 25 | T2720 | -10.262 |
| 6 | T4897 | -11.943 | 26 | T3795 | -10.260 |
| 7 | T4706 | -11.742 | 27 | T0148L | -10.222 |
| 8 | TMA2440 | -11.595 | 28 | T0853L | -10.211 |
| 9 | TMS1830 | -11.543 | 29 | T2133 | -10.121 |
| 10 | T4S1578 | -11.419 | 30 | T1723 | -10.095 |
| 11 | T2971 | -11.405 | 31 | T5022 | -10.049 |
| 12 | T0278 | -11.388 | 32 | T4718 | -9.996 |
| 13 | T2812 | -11.266 | 33 | T0966 | -9.958 |
| 14 | T2529 | -11.163 | 34 | T5075 | -9.942 |
| 15 | T4969 | -11.006 | 35 | T3910 | -9.899 |
| 16 | T1192 | -10.940 | 36 | T2727 | -9.898 |
| 17 | T5075 | -10.852 | 37 | T5022 | -9.866 |
| 18 | T4076 | -10.699 | 38 | T4901 | -9.856 |

| | | | | | |
|---|---|---|---|---|---|
| 19 | T1352 | -10.521 | 39 | T1658 | -9.837 |
| 20 | T2185 | -10.465 | 40 | T2001 | -9.814 |

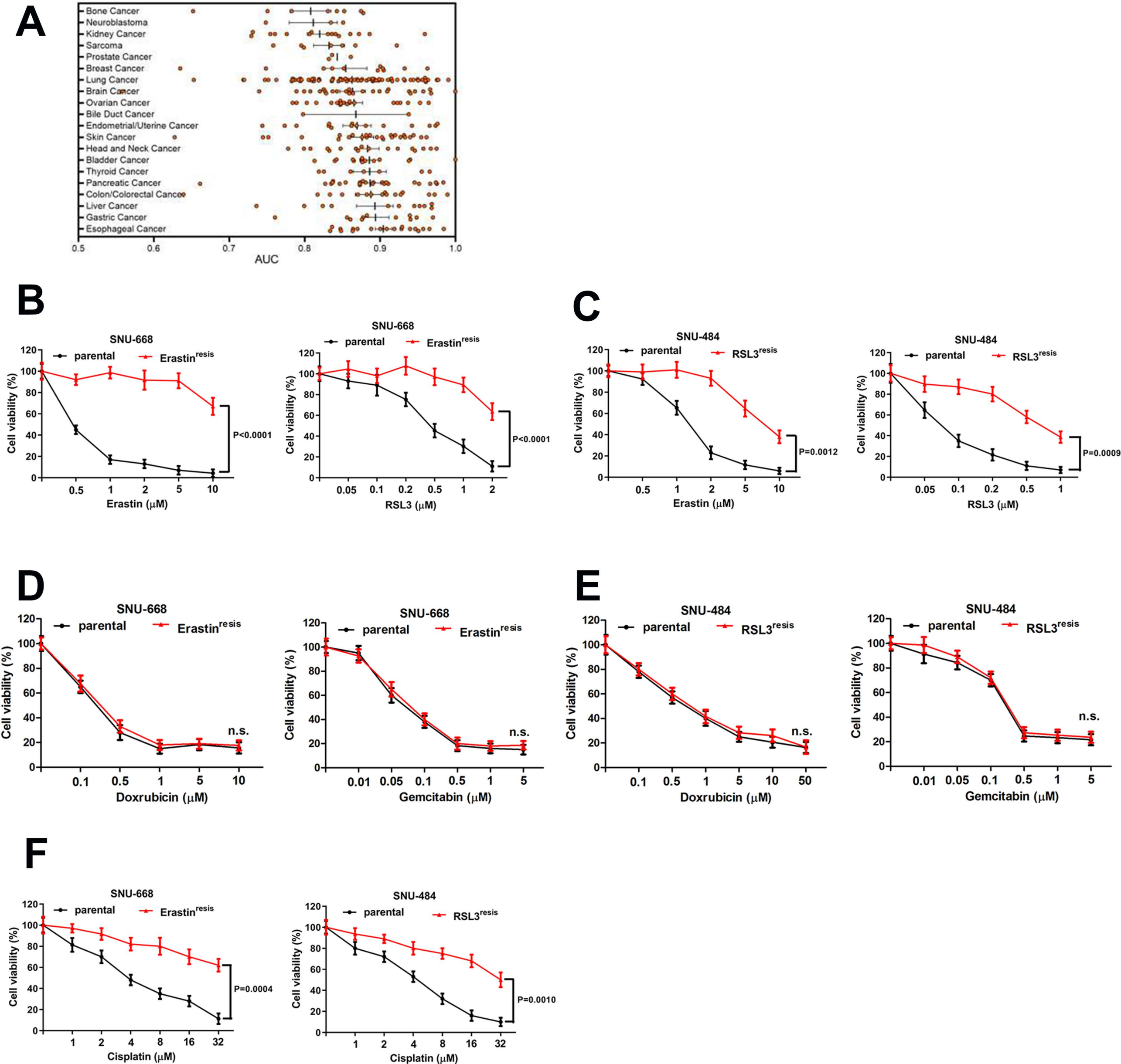
A
Bone Cancer
Neuroblastoma
Kidney Cancer
Sarcoma
Prostate Cancer
Breast Cancer
Lung Cancer
Brain Cancer
Ovarian Cancer
Bile Duct Cancer
Endometrial/Uterine Cancer
Skin Cancer
Head and Neck Cancer
Bladder Cancer
Thyroid Cancer
Pancreatic Cancer
Colon/Colorectal Cancer
Liver Cancer
Gastric Cancer
Esophageal Cancer
0.5 0.6 0.7 0.8 0.9 1.0
AUC
B
SNU-668
parental
Erastin^resis
Cell viability (%)
P<0.0001
Erastin (μM)
SNU-668
parental
Erastin^resis
P<0.0001
RSL3 (μM)
C
SNU-484
parental
RSL3^resis
P=0.0012
Erastin (μM)
SNU-484
parental
RSL3^resis
P=0.0009
RSL3 (μM)
D
SNU-668
parental
Erastin^resis
n.s.
Doxrubicin (μM)
SNU-668
parental
Erastin^resis
n.s.
Gemcitabin (μM)
E
SNU-484
parental
RSL3^resis
n.s.
Doxrubicin (μM)
SNU-484
parental
RSL3^resis
n.s.
Gemcitabin (μM)
F
SNU-668
parental
Erastin^resis
P=0.0004
Cisplatin (μM)
SNU-484
parental
RSL3^resis
P=0.0010
Cisplatin (μM)

**Supplementary Figure 1.** (A) The sensitivity of 20 tumor types to Erastin was analyzed using the DepMap database. AUC = Area Under Curve. Each dot represents a cell line from indicated cancer type. Cell lines with higher AUC values are more resistant to Erastin. (B,C) Relative viability of parental or Erastin$^{resis}$ SNU-668 cells (B) or RSL3$^{resis}$ SNU-484 (C) cells treated with different concentrations of the ferroptosis inducers Erastin or RSL3 for 24 h (n=3 independent experiments). Data are presented as mean values ± SD. (D,E) Relative viability of parental or Erastin$^{resis}$ SNU-668 cells (D) or RSL3$^{resis}$ SNU-484 (E) cells treated with different concentrations of the apoptosis inducers doxrubicin and gemcitabine for 24 h (n=3 independent experiments). Data are presented as mean values ± SD. (F) Relative viability of parental or Erastin$^{resis}$ SNU-668 cells or RSL3$^{resis}$ SNU-484 cells treated with different concentrations of cisplatin for 24 h. Cell viability was evaluated with a CellTiter-Glo luminescent cell viability assay (n=3 independent experiments). Data are presented as mean values ± SD. Statistical significance in (B-F) is determined by two-tailed unpaired *t*-test. Source data are provided as a Source Data file.

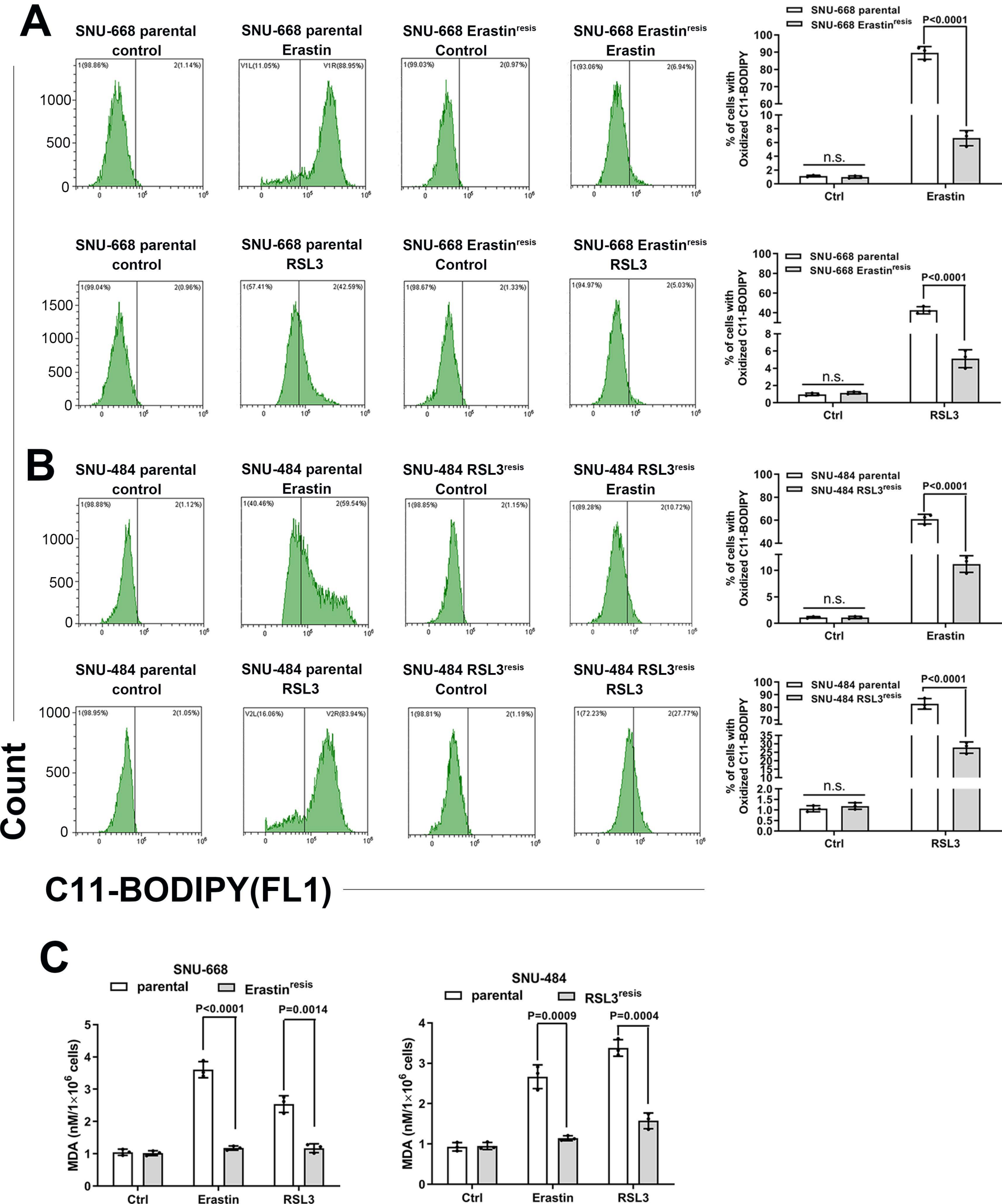

A
SNU-668 parental control
SNU-668 parental Erastin
SNU-668 Erastin^resis Control
SNU-668 Erastin^resis Erastin
SNU-668 parental
SNU-668 Erastin^resis
P<0.0001
n.s.
% of cells with Oxidized C11-BODIPY
Ctrl
Erastin
SNU-668 parental control
SNU-668 parental RSL3
SNU-668 Erastin^resis Control
SNU-668 Erastin^resis RSL3
RSL3
B
SNU-484 parental control
SNU-484 parental Erastin
SNU-484 RSL3^resis Control
SNU-484 RSL3^resis Erastin
SNU-484 parental
SNU-484 RSL3^resis
SNU-484 parental control
SNU-484 parental RSL3
SNU-484 RSL3^resis Control
SNU-484 RSL3^resis RSL3
Count
C11-BODIPY(FL1)
C
SNU-668
parental
Erastin^resis
P<0.0001
P=0.0014
MDA (nM/1×10^6 cells)
SNU-484
RSL3^resis
P=0.0009
P=0.0004

**Supplementary Figure 2.** (A-C) Parental or Erastin$^{resis}$ SNU-668 cells or RSL3$^{resis}$ SNU-484 cells treated with Erastin (0, 2 μM) or RSL3 (0, 0.5 μM) for 24 h (n=3 independent experiments). (A,B) Representative histogram plot for fluorescence of oxidized BODIPY-C11. (C) Intracellular MDA were assayed with ELISA. Data are presented as mean values ± SD. Statistical significance in (A-C) is determined by two-tailed unpaired *t*-test. Source data are provided as a Source Data file.

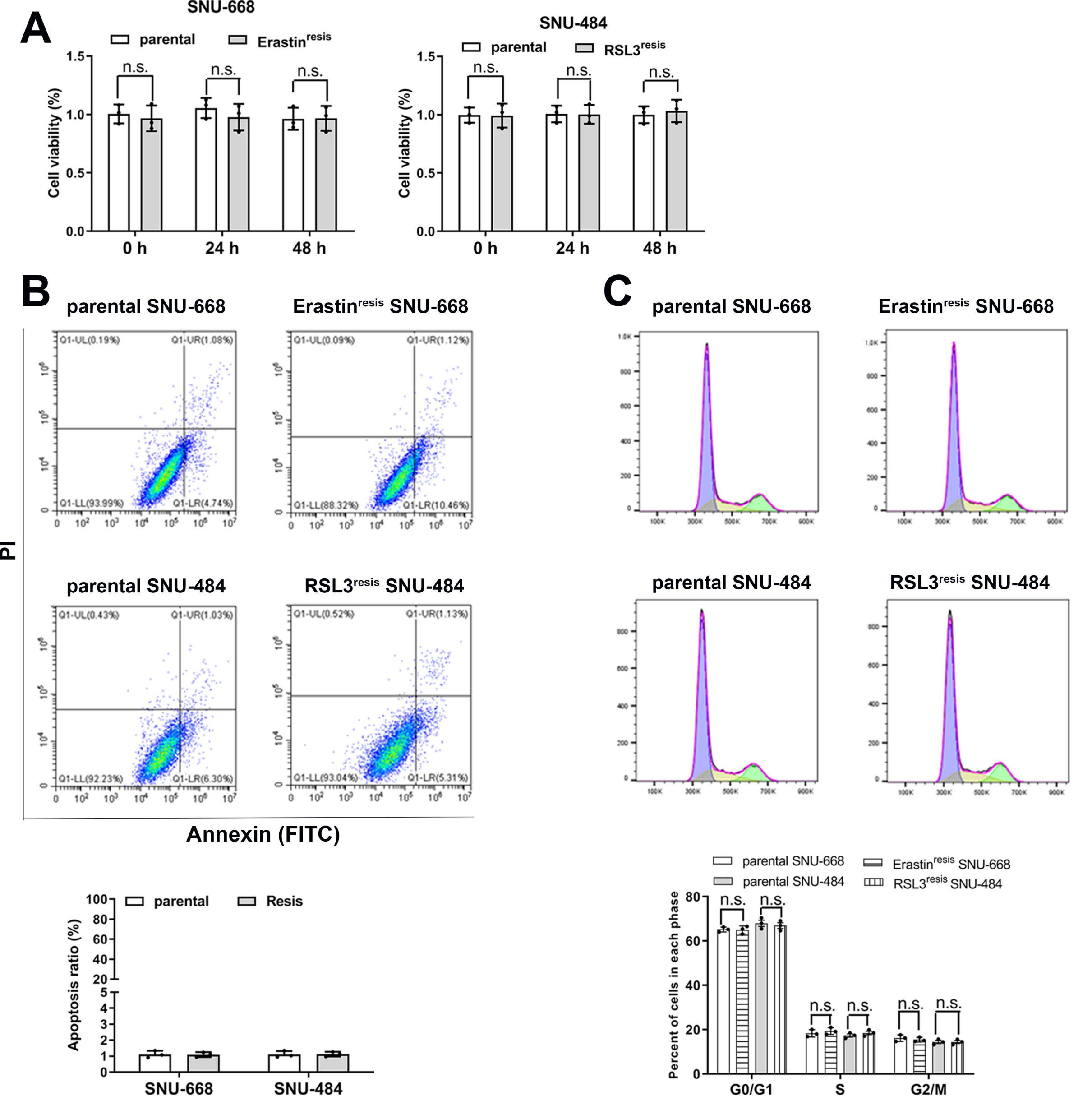
A
SNU-668
parental
Erastin resis
Cell viability (%)
n.s.
0 h
24 h
48 h
SNU-484
RSL3 resis
B
parental SNU-668
Erastin resis SNU-668
parental SNU-484
RSL3 resis SNU-484
PI
Annexin (FITC)
Q1-UL(0.19%)
Q1-UR(1.08%)
Q1-LL(93.99%)
Q1-LR(4.74%)
Q1-UL(0.09%)
Q1-UR(1.12%)
Q1-LL(88.32%)
Q1-LR(10.46%)
Q1-UL(0.43%)
Q1-UR(1.03%)
Q1-LL(92.23%)
Q1-LR(6.30%)
Q1-UL(0.52%)
Q1-UR(1.13%)
Q1-LL(93.04%)
Q1-LR(5.31%)
Apoptosis ratio (%)
parental
Resis
SNU-668
SNU-484
C
parental SNU-668
Erastin resis SNU-668
parental SNU-484
RSL3 resis SNU-484
parental SNU-668
Erastin resis SNU-668
parental SNU-484
RSL3 resis SNU-484
Percent of cells in each phase
G0/G1
S
G2/M

**Supplementary Figure 3.** (A) Relative viability of parental or Erastin$^{resis}$ SNU-668 cells or RSL3$^{resis}$ SNU-484 cells cultured after 24 h or 48 h (n=3 independent experiments). (B) Annexin V/PI staining and flow cytometry analysis assessing apoptosis in parental or Erastin$^{resis}$ SNU-668 cells or RSL3$^{resis}$ SNU-484 cells (n=3 independent experiments). (C) Cell cycle analysis in parental or Erastin$^{resis}$ SNU-668 cells or RSL3$^{resis}$ SNU-484 cells determined the relative cell numbers in each cell-cycle phase with propidium iodide staining (n=3 independent experiments). Statistical significance in (A-C) is determined by two-tailed unpaired *t*-test. Data are presented as mean values ± SD. Source data are provided as a Source Data file.

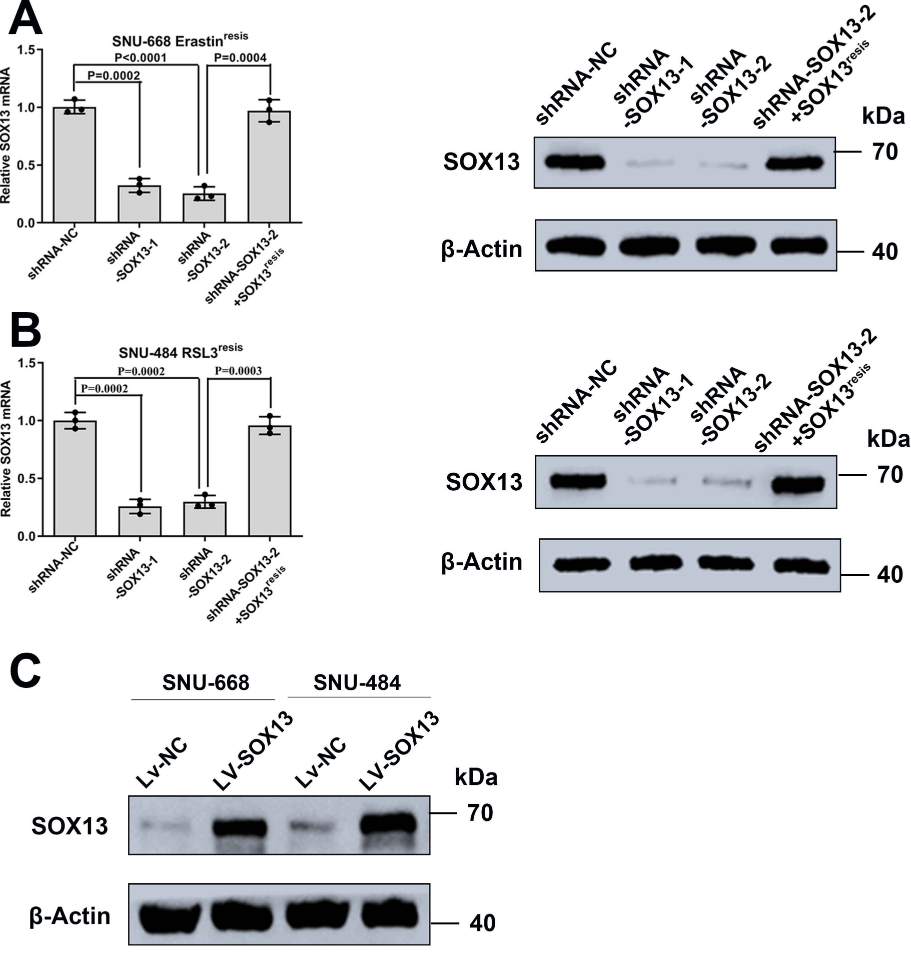
A
SNU-668 Erastin^resis
P<0.0001
P=0.0002
P=0.0004
Relative SOX13 mRNA
1.5
1.0
0.5
0.0
shRNA-NC
shRNA -SOX13-1
shRNA -SOX13-2
shRNA-SOX13-2 +SOX13^resis
kDa
SOX13
70
β-Actin
40
B
SNU-484 RSL3^resis
P=0.0002
P=0.0002
P=0.0003
C
SNU-668
SNU-484
Lv-NC
LV-SOX13
Lv-NC
LV-SOX13

**Supplementary Figure 4.** SOX13 expression was detected in Erastin$^{resis}$ SNU-668 cells (A) or RSL3$^{resis}$ SNU-484 cells (B) by western blot after transduction of lentiviruses encoding SOX13 shRNA-1 or shRNA-2 or a scrambled shRNA and/or resistant SOX13 cDNA (SOX13$^{resis}$) (n=3 independent experiments). Data are presented as mean values ± SD. (C) SOX13 expression was detected in parental SNU-668 and SNU-484 cells by western blot after transfection of lentivirus harboring the human SOX13 sequence or the empty vector (n=3 independent experiments). Statistical significance in (A,B) is determined by two-tailed unpaired *t*-test. Source data are provided as a Source Data file.

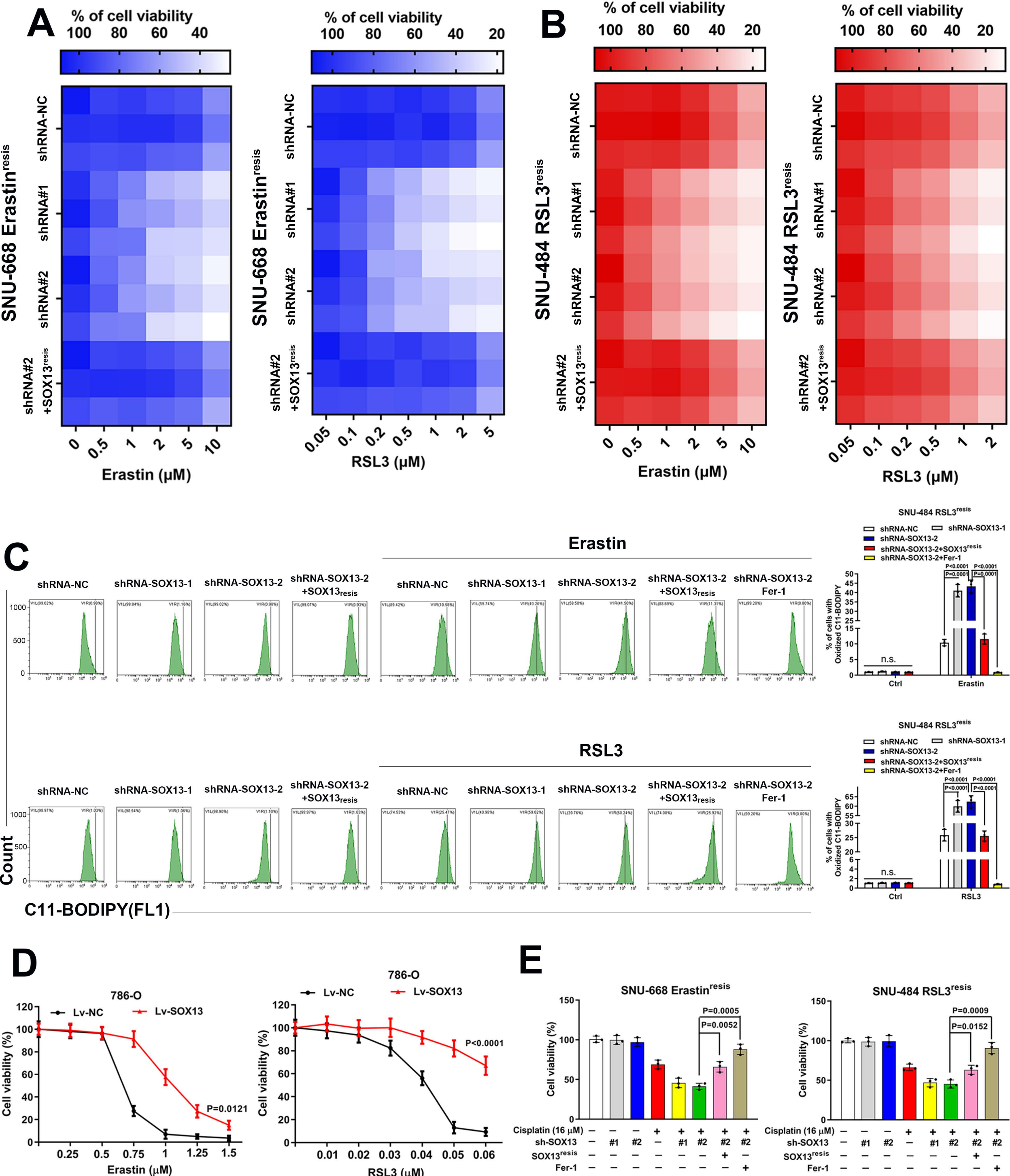
A
% of cell viability
SNU-668 Erastin$^{resis}$
shRNA-NC
shRNA#1
shRNA#2
shRNA#2 +SOX13$^{resis}$
Erastin (μM)
SNU-668 Erastin$^{resis}$
RSL3 (μM)
B
SNU-484 RSL3$^{resis}$
Erastin (μM)
RSL3 (μM)
C
Erastin
RSL3
shRNA-NC
shRNA-SOX13-1
shRNA-SOX13-2
shRNA-SOX13-2 +SOX13resis
shRNA-SOX13-2 Fer-1
Count
C11-BODIPY(FL1)
SNU-484 RSL3$^{resis}$
shRNA-SOX13-2+SOX13$^{resis}$
shRNA-SOX13-2+Fer-1
% of cells with Oxidized C11-BODIPY
P<0.0001
n.s.
Ctrl
D
786-O
Lv-NC
Lv-SOX13
Cell viability (%)
P=0.0121
P<0.0001
Erastin (μM)
RSL3 (μM)
E
SNU-668 Erastin$^{resis}$
SNU-484 RSL3$^{resis}$
P=0.0005
P=0.0052
P=0.0009
P=0.0152
Cisplatin (16 μM)
sh-SOX13
SOX13$^{resis}$
Fer-1

**Supplementary Figure 5.** (A-C) Effect of knockdown of SOX13 and SOX13 re-expression on sensitivity of $Erastin^{resis}$ SNU-668 cells (A) or $RSL3^{resis}$ SNU-484 cells (B) to the ferroptosis inducers RSL3 or Erastin. The heatmap shows altered cell viability. Cells were treated with lentiviruses encoding SOX13 shRNA-1 or shRNA-2 or a scrambled shRNA alone and/or resistant SOX13 cDNA ($SOX13^{resis}$), or in combination with various concentrations of RSL3 or Erastin for 24 hours. The heatmap shows altered cell viability. (C) SNU-484 $RSL3^{resis}$ cells were treated with Erastin (2 μM) or RSL3 (0.5 μM) for 24 hours. Lipid peroxidation was determined with a lipid peroxidation C11-BODIPY assay in SNU-484 $RSL3^{resis}$ cells (n=3 independent experiments), and a representative flow cytometry histogram plot is presented. Data are presented as mean values ± SD. (D) 786-O cells were treated with lentivirus harboring the human SOX13 sequence or the empty vector. Cells were treated with different concentrations of the ferroptosis inducers Erastin or RSL3 for 24 h (n=3 independent experiments). Data are presented as mean values ± SD. (E) Effect of SOX13 downregulation on cisplatin sensitivity in the absence or presence of Fer-1 (1 μM) in $Erastin^{resis}$ SNU-668 cells or $RSL3^{resis}$ SNU-484 cells (n=3 independent experiments). The cells were treated with cisplatin (16 μM) for 24 hours. Data are presented as mean values ± SD. Statistical significance in (C-E) is determined by two-tailed unpaired *t*-test. Source data are provided as a Source Data file.

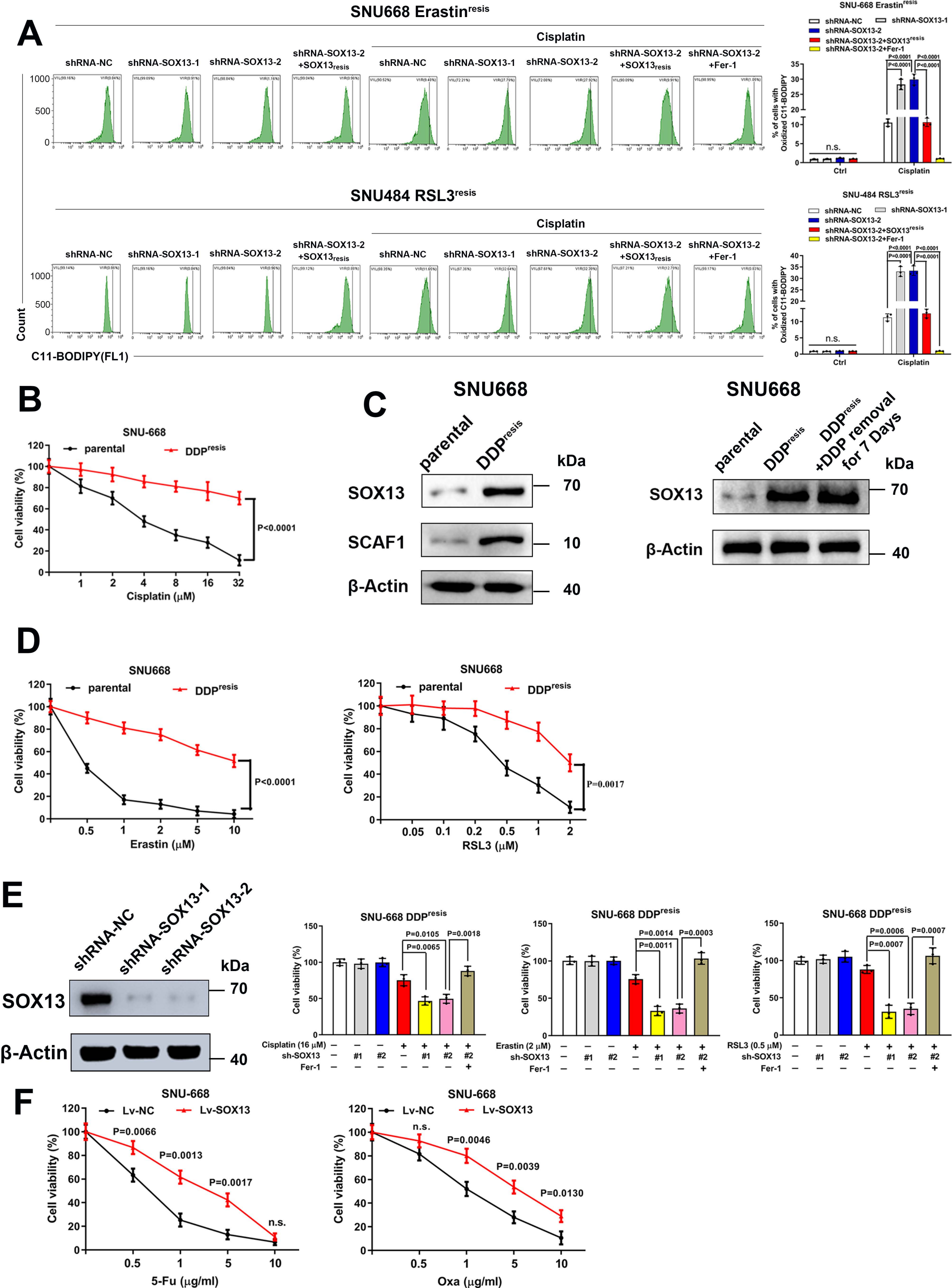

A
SNU668 Erastin resis
Cisplatin
shRNA-NC
shRNA-SOX13-1
shRNA-SOX13-2
shRNA-SOX13-2 +SOX13resis
shRNA-SOX13-2 +Fer-1
SNU484 RSL3 resis
Count
C11-BODIPY(FL1)
SNU-668 Erastin resis
SNU-484 RSL3 resis
% of cells with Oxidized C11-BODIPY
n.s.
Ctrl
P<0.0001
P=0.0001
B
SNU-668
parental
DDP resis
Cell viability (%)
Cisplatin (μM)
P<0.0001
C
SNU668
parental
DDP resis
DDP resis +DDP removal for 7 Days
kDa
SOX13
SCAF1
β-Actin
70
10
40
D
SNU668
Erastin (μM)
RSL3 (μM)
P=0.0017
E
shRNA-NC
shRNA-SOX13-1
shRNA-SOX13-2
SNU-668 DDP resis
Cisplatin (16 μM)
Erastin (2 μM)
RSL3 (0.5 μM)
sh-SOX13
Fer-1
P=0.0105
P=0.0065
P=0.0018
P=0.0014
P=0.0011
P=0.0003
P=0.0006
P=0.0007
F
SNU-668
Lv-NC
Lv-SOX13
P=0.0066
P=0.0013
P=0.0017
n.s.
5-Fu (μg/ml)
P=0.0046
P=0.0039
P=0.0130
Oxa (μg/ml)

**Supplementary Figure 6.** (A) Effect of SOX13 downregulation and SOX13 re-expression on cisplatin (16 μM) sensitivity in the absence or presence of Fer-1 (1 μM) in SNU-668 Erastin$^{resis}$ and SNU-484 RSL3$^{resis}$ cells. Cells were treated with cisplatin (16 μM) for 24 hours. Lipid peroxidation was determined with a lipid peroxidation C11-BODIPY assay (n=3 independent experiments) and a representative flow cytometry histogram plot is presented. Data are presented as mean values ± SD. (B) Relative viability of parental or DDP$^{resis}$ SNU-668 cells treated with different concentrations of cisplatin for 24 h (n=3 independent experiments). Data are presented as mean values ± SD. (C) SOX13 and SCAF1 were determined by immunoblotting assay and normalized to β-actin (n=3 independent experiments). (D) Relative viability of parental or DDP$^{resis}$ SNU-668 cells treated with different concentrations of Erastin or RSL3 for 24 h (n=3 independent experiments). Data are presented as mean values ± SD. (E) Effect of SOX13 downregulation on Erastin (2 μM), RSL3 (0.5 μM) or Cisplatin (16 μM) sensitivity in the absence or presence of Fer-1 (1 μM) in DDP$^{resis}$ SNU-668 cells (n=3 independent experiments). The cells were treated with FINs or cisplatin for 24 hours. Data are presented as mean values ± SD. (F) SNU-668 cells were treated with lentivirus harboring the human SOX13 sequence or the empty vector. Cells were treated with different concentrations of 5-Fu or oxaliplatin for 24 h. Cell viability was evaluated with a CellTiter-Glo luminescent cell viability assay (n=3 independent experiments). Data are presented as mean values ± SD. Statistical significance in (A,B,D-F) is determined by two-tailed unpaired *t*-test. Source data are provided as a Source Data file.

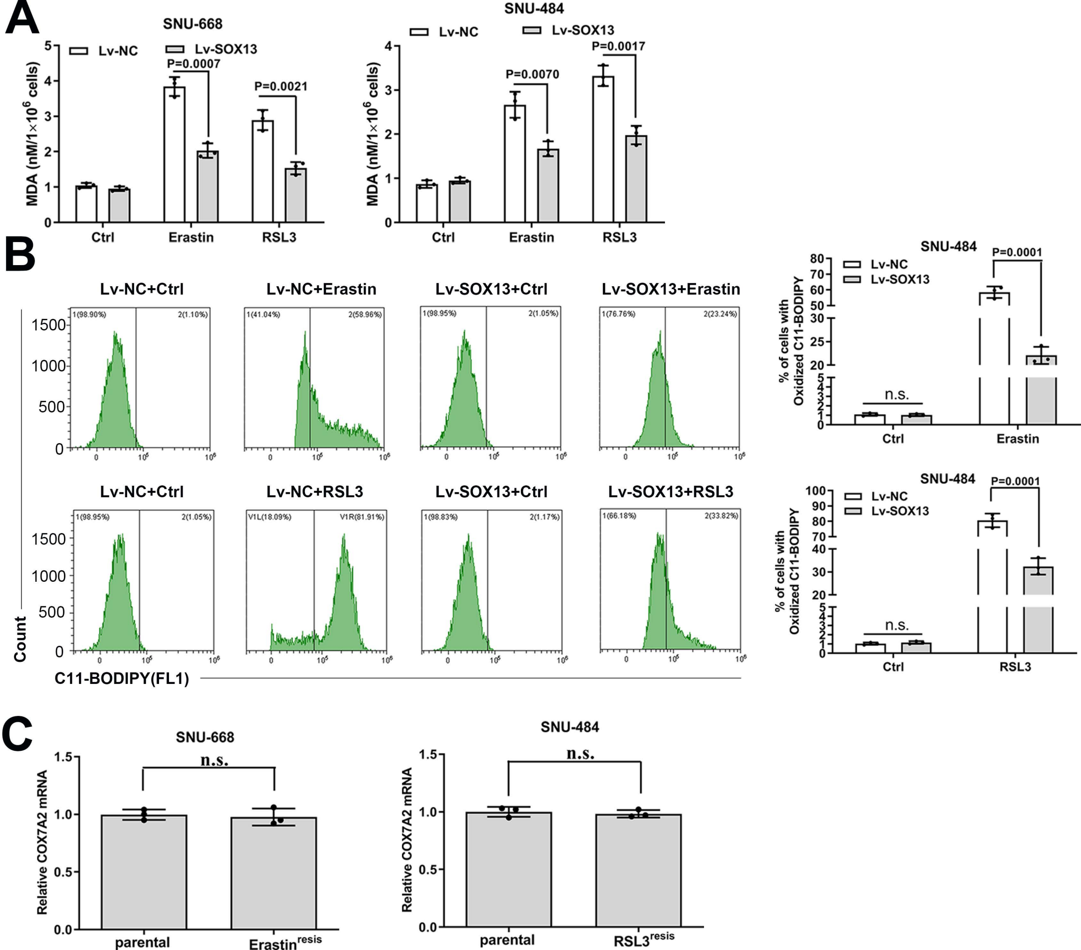

A
SNU-668
Lv-NC
Lv-SOX13
P=0.0007
P=0.0021
MDA (nM/1×10^6 cells)
Ctrl
Erastin
RSL3
SNU-484
P=0.0070
P=0.0017
B
Lv-NC+Ctrl
Lv-NC+Erastin
Lv-SOX13+Ctrl
Lv-SOX13+Erastin
Lv-NC+RSL3
Lv-SOX13+RSL3
Count
C11-BODIPY(FL1)
% of cells with Oxidized C11-BODIPY
P=0.0001
n.s.
C
Relative COX7A2 mRNA
parental
Erastin^resis
RSL3^resis

**Supplementary Figure 7.** (A,B) SNU-668/SNU-484 Cells were treated with lentivirus harboring the human SOX13 sequence or the empty vector, or in combination with Erastin (2 μM) or RSL3 (0.5 μM) for 24 hours. (A) ELISA was utilized to assess the intracellular MDA level (n=3 independent experiments). Data are presented as mean values ± SD. (B) Lipid peroxidation was determined with a lipid peroxidation C11-BODIPY assay (n=3 independent experiments), and a representative flow cytometry histogram plot is presented. Data are presented as mean values ± SD. (C) COX7A2 mRNA abundance in resistant versus parental cell lines of SNU-668 and SNU-484 (n=3 independent experiments). Data are presented as mean values ± SD. Statistical significance in (A-C) is determined by two-tailed unpaired *t*-test. Source data are provided as a Source Data file.

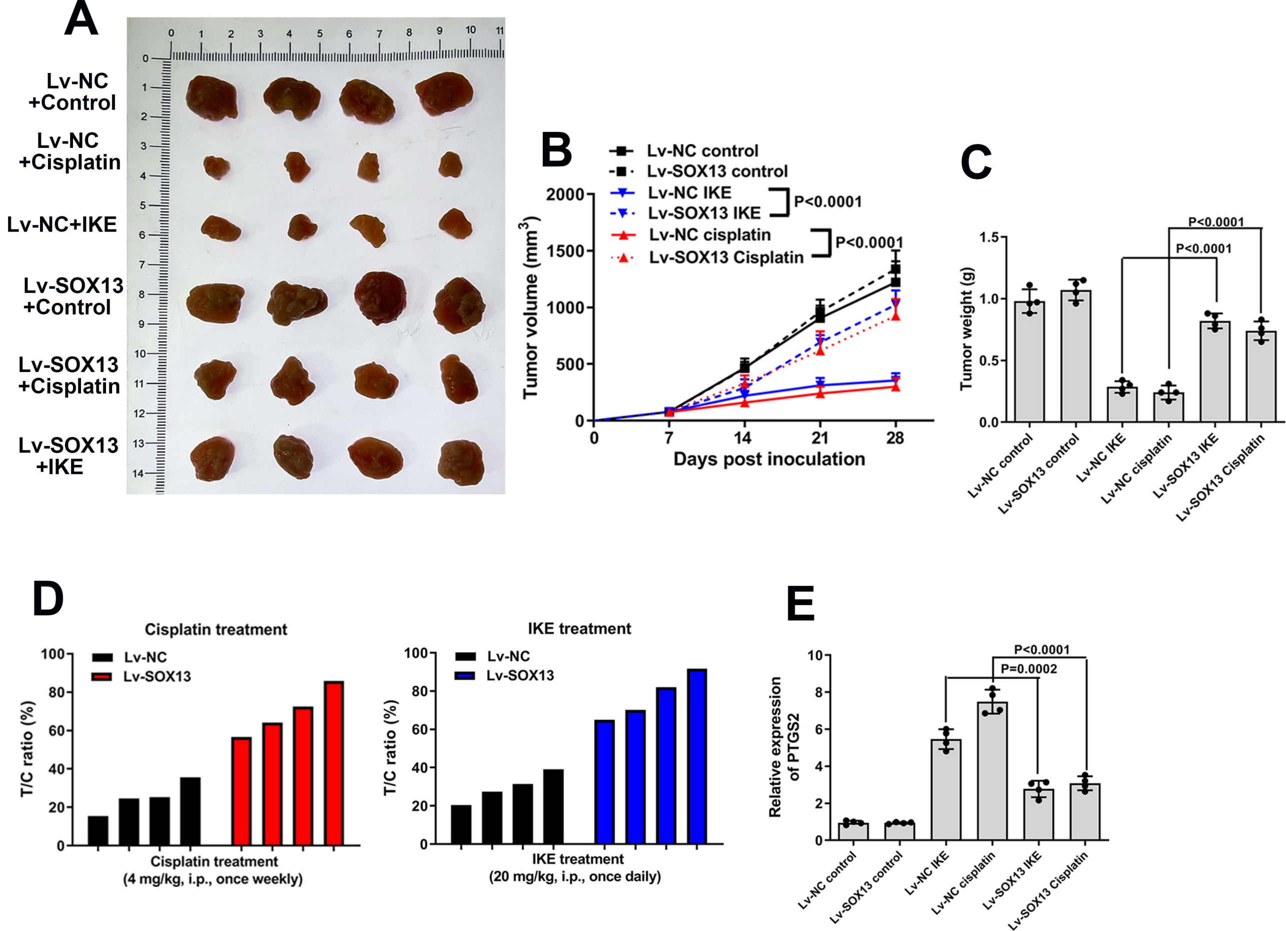

A
Lv-NC +Control
Lv-NC +Cisplatin
Lv-NC+IKE
Lv-SOX13 +Control
Lv-SOX13 +Cisplatin
Lv-SOX13 +IKE
B
Lv-NC control
Lv-SOX13 control
Lv-NC IKE
Lv-SOX13 IKE
P<0.0001
Lv-NC cisplatin
Lv-SOX13 Cisplatin
P<0.0001
Tumor volume (mm3)
Days post inoculation
C
Tumor weight (g)
P<0.0001
P<0.0001
Lv-NC control
Lv-SOX13 control
Lv-NC IKE
Lv-NC cisplatin
Lv-SOX13 IKE
Lv-SOX13 Cisplatin
D
Cisplatin treatment
Lv-NC
Lv-SOX13
T/C ratio (%)
Cisplatin treatment
(4 mg/kg, i.p., once weekly)
IKE treatment
Lv-NC
Lv-SOX13
T/C ratio (%)
IKE treatment
(20 mg/kg, i.p., once daily)
E
Relative expression of PTGS2
P<0.0001
P=0.0002
Lv-NC control
Lv-SOX13 control
Lv-NC IKE
Lv-NC cisplatin
Lv-SOX13 IKE
Lv-SOX13 Cisplatin

**Supplementary Figure 8.** SNU-484-SOX13-CDX tumors and SNU-484-NC-CDX tumors were treated with cisplatin and IKE. Representative images of tumors formed (A), tumor growth curves (B), tumor weights (C), T/C ratio (D) and PTGS2 (E) expression analysis are shown. Treatment with IKE (20 mg/kg, i.p., once daily), cisplatin (4 mg/kg, i.p., once weekly) or PBS (100 μl, i.p., once daily) started on Day 7 and lasted for 3 consecutive weeks (n=4 mice per group). T/C% = $T_{RTV}/C_{RTV} \times 100\%$; $T_{RTV}$, relative tumor volume after treatment; $C_{RTV}$, relative tumor volume of control group. Data are presented as mean values ± SD. Statistical significance in (B,C,E) is determined by two-tailed unpaired *t*-test. Source data are provided as a Source Data file.

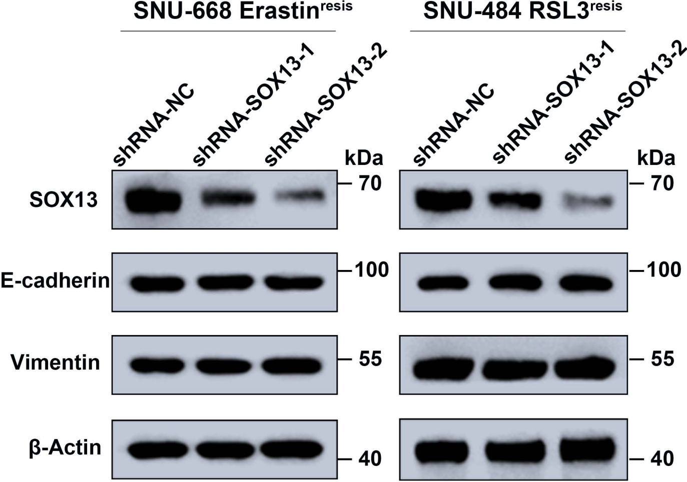
SNU-668 Erastin^resis
SNU-484 RSL3^resis
shRNA-NC
shRNA-SOX13-1
shRNA-SOX13-2
shRNA-NC
shRNA-SOX13-1
shRNA-SOX13-2
kDa
kDa
SOX13
E-cadherin
Vimentin
β-Actin
70
100
55
40
70
100
55
40

**Supplementary Figure 9.** Effect of SOX13 knockdown on E-cadherin and Viementin protein abundance in FINs resistant cells (n=3 independent experiments).

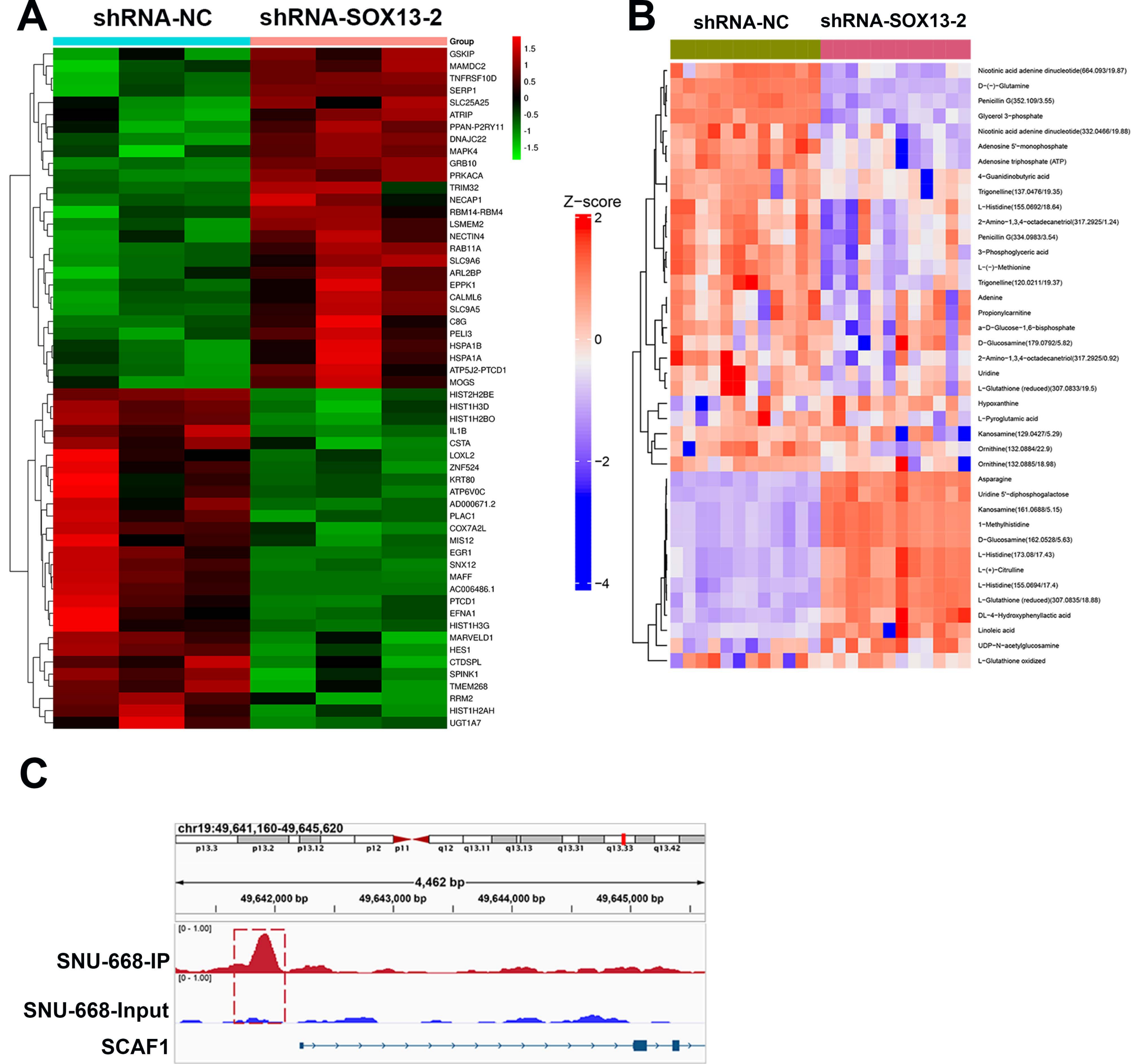

A
shRNA-NC
shRNA-SOX13-2
Group
1.5
1
0.5
0
-0.5
-1
-1.5
GSKIP
MAMDC2
TNFRSF10D
SERP1
SLC25A25
ATRIP
PPAN-P2RY11
DNAJC22
MAPK4
GRB10
PRKACA
TRIM32
NECAP1
RBM14-RBM4
LSMEM2
NECTIN4
RAB11A
SLC9A6
ARL2BP
EPPK1
CALML6
SLC9A5
C8G
PELI3
HSPA1B
HSPA1A
ATP5J2-PTCD1
MOGS
HIST2H2BE
HIST1H3D
HIST1H2BO
IL1B
CSTA
LOXL2
ZNF524
KRT80
ATP6V0C
AD000671.2
PLAC1
COX7A2L
MIS12
EGR1
SNX12
MAFF
AC006486.1
PTCD1
EFNA1
HIST1H3G
MARVELD1
HES1
CTDSPL
SPINK1
TMEM268
RRM2
HIST1H2AH
UGT1A7
Z−score
2
0
−2
−4
B
shRNA-NC
shRNA-SOX13-2
Nicotinic acid adenine dinucleotide(664.093/19.87)
D−(−)−Glutamine
Penicillin G(352.109/3.55)
Glycerol 3−phosphate
Nicotinic acid adenine dinucleotide(332.0466/19.88)
Adenosine 5'−monophosphate
Adenosine triphosphate (ATP)
4−Guanidinobutyric acid
Trigonelline(137.0476/19.35)
L−Histidine(155.0692/18.64)
2−Amino−1,3,4−octadecanetriol(317.2925/1.24)
Penicillin G(334.0983/3.54)
3−Phosphoglyceric acid
L−(−)−Methionine
Trigonelline(120.0211/19.37)
Adenine
Propionylcarnitine
a−D−Glucose−1,6−bisphosphate
D−Glucosamine(179.0792/5.82)
2−Amino−1,3,4−octadecanetriol(317.2925/0.92)
Uridine
L−Glutathione (reduced)(307.0833/19.5)
Hypoxanthine
L−Pyroglutamic acid
Kanosamine(129.0427/5.29)
Ornithine(132.0884/22.9)
Ornithine(132.0885/18.98)
Asparagine
Uridine 5'−diphosphogalactose
Kanosamine(161.0688/5.15)
1−Methylhistidine
D−Glucosamine(162.0528/5.63)
L−Histidine(173.08/17.43)
L−(+)−Citrulline
L−Histidine(155.0694/17.4)
L−Glutathione (reduced)(307.0835/18.88)
DL−4−Hydroxyphenyllactic acid
Linoleic acid
UDP−N−acetylglucosamine
L−Glutathione oxidized
C
chr19:49,641,160-49,645,620
p13.3
p13.2
p13.12
p12
p11
q12
q13.11
q13.13
q13.31
q13.33
q13.42
4,462 bp
49,642,000 bp
49,643,000 bp
49,644,000 bp
49,645,000 bp
[0 - 1.00]
SNU-668-IP
[0 - 1.00]
SNU-668-Input
SCAF1

**Supplementary Figure 10.** (A) Heatmap of the top 56 dysregulated genes (>3-fold; $p < 0.05$) in response to SOX13 knockdown (n=3 independent experiments). (B) Metabolic profile changes from control to SOX13-downregulated Erastin$^{resis}$ SNU-668 cells (n= 12 independent experiments). (C) Binding profiles and peak calling records of SOX13 in the SCAF1 promoter. Significant peak ($P < 1 \times 10^{-11}$) is highlighted by a red box with dotted line.

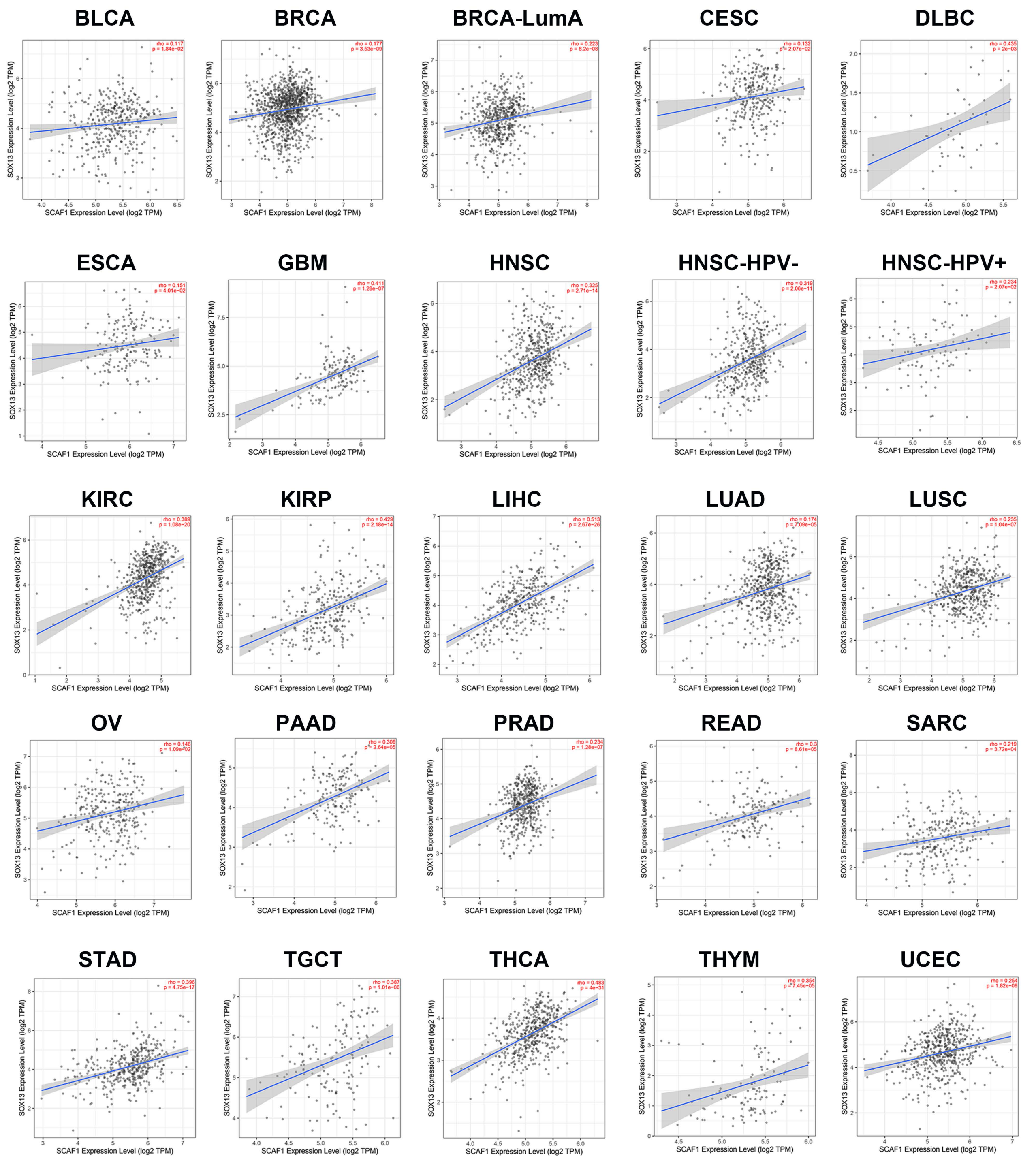
BLCA
rho = 0.117
p = 1.84e-02
BRCA
rho = 0.177
p = 3.53e-09
BRCA-LumA
rho = 0.223
p = 8.2e-08
CESC
rho = 0.132
p = 2.07e-02
DLBC
rho = 0.435
p = 2e-03
ESCA
rho = 0.151
p = 4.01e-02
GBM
rho = 0.411
p = 1.28e-07
HNSC
rho = 0.325
p = 2.71e-14
HNSC-HPV-
rho = 0.319
p = 2.06e-11
HNSC-HPV+
rho = 0.234
p = 2.07e-02
KIRC
rho = 0.389
p = 1.08e-20
KIRP
rho = 0.429
p = 2.18e-14
LIHC
rho = 0.513
p = 2.67e-26
LUAD
rho = 0.174
p = 7.09e-05
LUSC
rho = 0.235
p = 1.04e-07
OV
rho = 0.146
p = 1.09e-02
PAAD
rho = 0.309
p = 2.64e-05
PRAD
rho = 0.234
p = 1.28e-07
READ
rho = 0.3
p = 8.61e-05
SARC
rho = 0.219
p = 3.72e-04
STAD
rho = 0.396
p = 4.75e-17
TGCT
rho = 0.387
p = 1.01e-06
THCA
rho = 0.483
p = 4e-31
THYM
rho = 0.354
p = 7.45e-05
UCEC
rho = 0.254
p = 1.82e-09
SOX13 Expression Level (log2 TPM)
SCAF1 Expression Level (log2 TPM)

**Supplementary Figure 11.** Scatterplots of correlations between SOX13 expression and SCAF1 expression adjusted by purity according to TIMER (http://cistrome.org/TIMER/) in BLCA (bladder urothelial carcinoma, n=408), BRCA (breast invasive carcinoma, n=1100), BRCA-LumA (breast invasive carcinoma luminal A type, n=568), CESC (Cervical squamous cell carcinoma and endocervical adenocarcinoma, n=306), DLBC (Lymphoid Neoplasm Diffuse Large B-cell Lymphoma, n=48), ESCA (Esophageal carcinoma, n=185), GBM (Glioblastoma multiforme, n=153), HNSC (Head and Neck squamous cell carcinoma, n=522), HNSC-HPV- (Head and Neck squamous cell carcinoma HPV negative, n=422), HNSC-HPV+ (Head and Neck squamous cell carcinoma HPV positive, n=98), KIRC (Kidney renal clear cell carcinoma, n=533), KIRP (Kidney renal papillary cell carcinoma, n=290), LIHC (Liver hepatocellular carcinoma, n=371), LUAD (Lung adenocarcinoma, n=515), LUSC (Lung squamous cell carcinoma, n=501), OV (Ovarian serous cystadenocarcinoma, n=303), PAAD (Pancreatic adenocarcinoma, n=179), PRAD (Prostate adenocarcinoma, n=498), READ (Rectum adenocarcinoma, n=166), SARC (Sarcoma, n=260), STAD (Stomach adenocarcinoma, n=415), TGCT (Testicular Germ Cell Tumors, n=150), THCA (Thyroid carcinoma, n=509), THYM (Thymoma, n=120), UCEC (Uterine Corpus Endometrial Carcinoma, n=545).

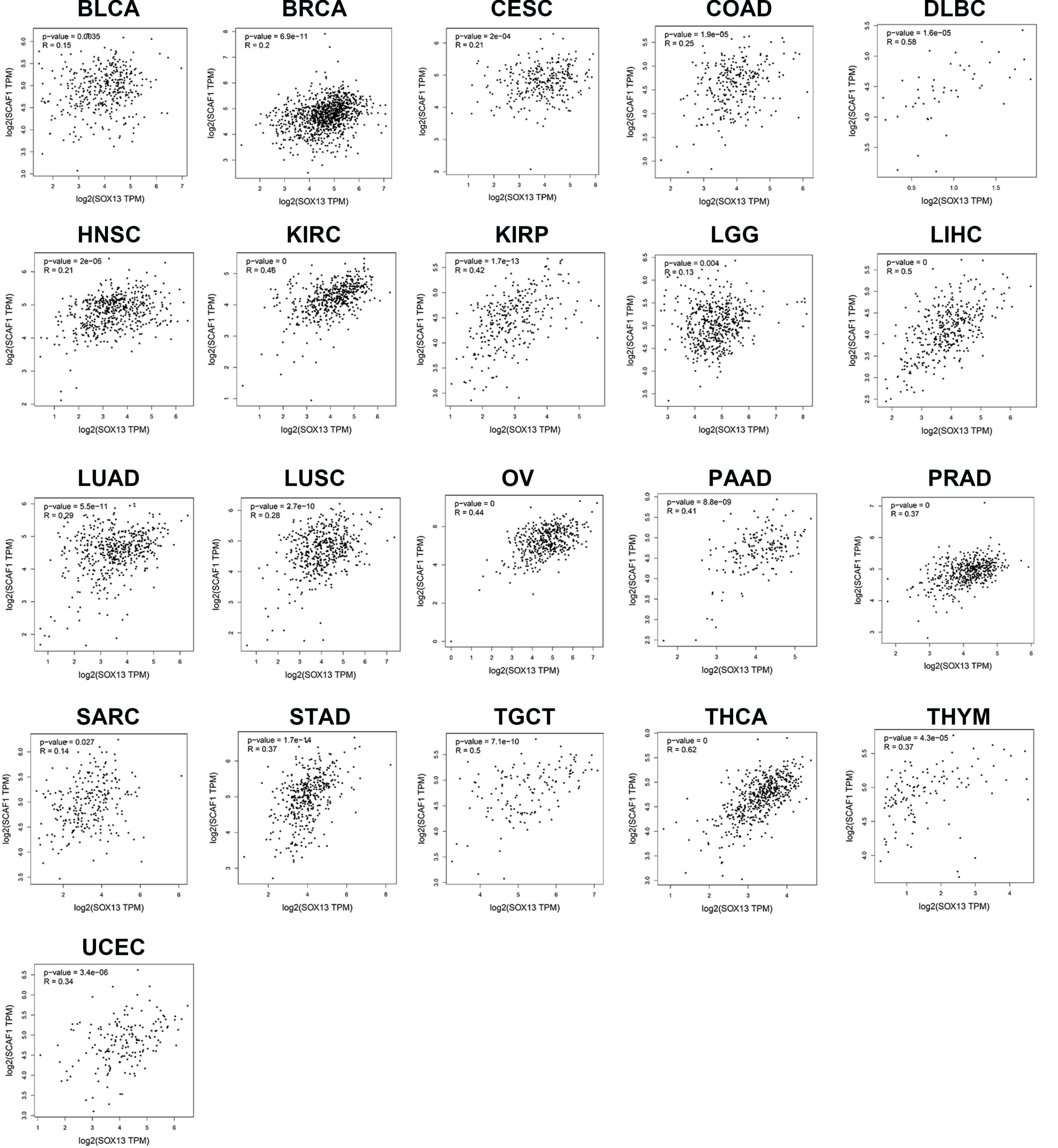
BLCA
p-value = 0.0035
R = 0.15
BRCA
p-value = 6.9e-11
R = 0.2
CESC
p-value = 2e-04
R = 0.21
COAD
p-value = 1.9e-05
R = 0.25
DLBC
p-value = 1.6e-05
R = 0.58
HNSC
p-value = 2e-06
R = 0.21
KIRC
p-value = 0
R = 0.46
KIRP
p-value = 1.7e-13
R = 0.42
LGG
p-value = 0.004
R = 0.13
LIHC
p-value = 0
R = 0.5
LUAD
p-value = 5.5e-11
R = 0.29
LUSC
p-value = 2.7e-10
R = 0.28
OV
p-value = 0
R = 0.44
PAAD
p-value = 8.8e-09
R = 0.41
PRAD
p-value = 0
R = 0.37
SARC
p-value = 0.027
R = 0.14
STAD
p-value = 1.7e-14
R = 0.37
TGCT
p-value = 7.1e-10
R = 0.5
THCA
p-value = 0
R = 0.62
THYM
p-value = 4.3e-05
R = 0.37
UCEC
p-value = 3.4e-06
R = 0.34
log2(SCAF1 TPM)
log2(SOX13 TPM)

**Supplementary Figure 12.** Correlation results between SOX13 and SCAF1 according to GEPIA (http://gepia.cancer-pku.cn/) in BLCA, BRCA, CESC, COAD, DLBC, HNSC, KIRC, KIRP, LGG (Brain Lower Grade Glioma), LIHC, LUAD, LUSC, OV, PAAD, PRAD, SARC, STAD, TGCT, THCA, THYM, UCEC.

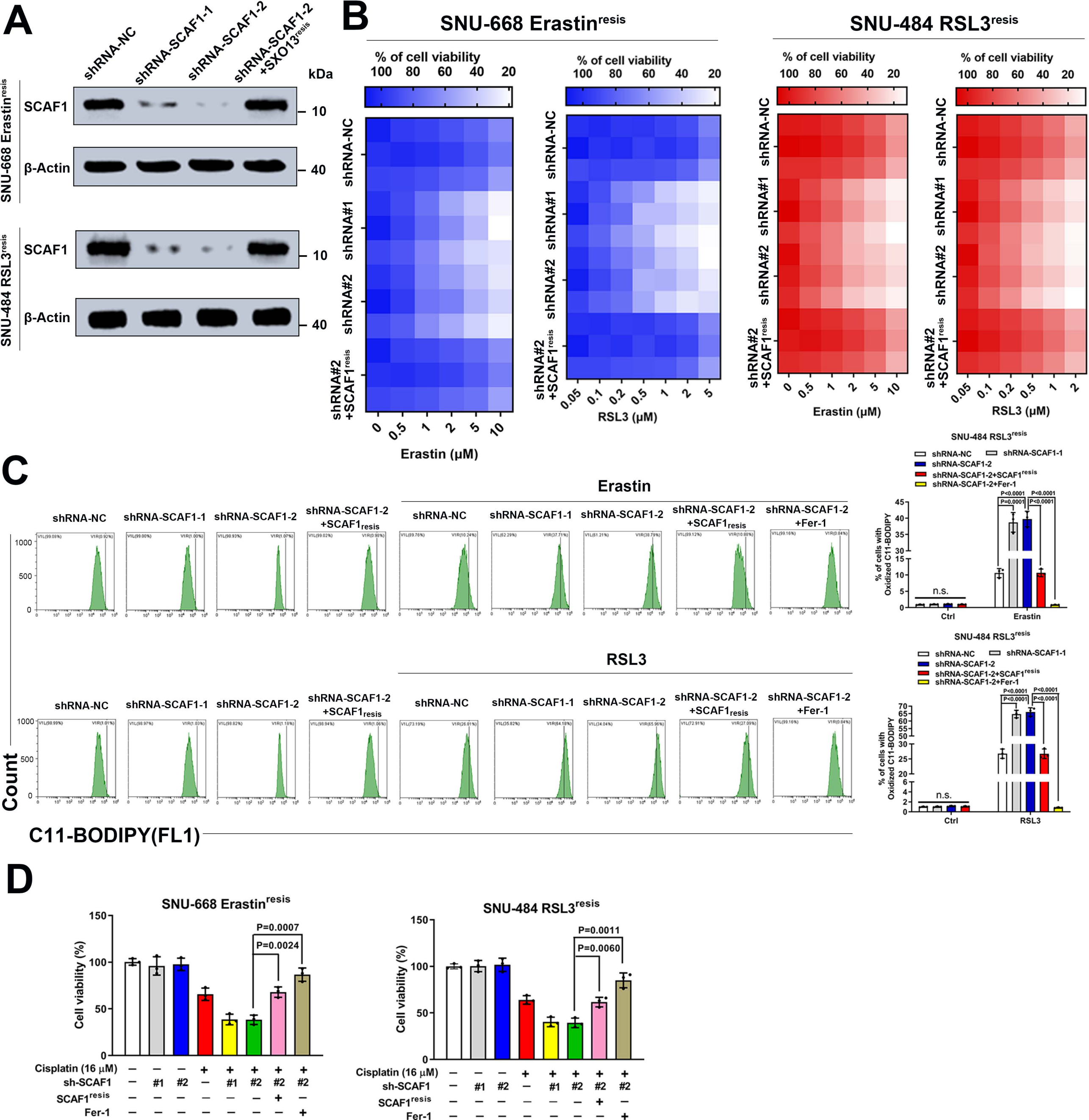

A
shRNA-NC
shRNA-SCAF1-1
shRNA-SCAF1-2
shRNA-SCAF1-2 +SXO13resis
kDa
SNU-668 Erastinresis
SNU-484 RSL3resis
SCAF1
β-Actin
10
40
B
SNU-668 Erastinresis
SNU-484 RSL3resis
% of cell viability
100 80 60 40 20
shRNA-NC
shRNA#1
shRNA#2
shRNA#2 +SCAF1resis
Erastin (μM)
RSL3 (μM)
C
Erastin
RSL3
shRNA-NC
shRNA-SCAF1-1
shRNA-SCAF1-2
shRNA-SCAF1-2 +SCAF1resis
shRNA-SCAF1-2 +Fer-1
Count
C11-BODIPY(FL1)
SNU-484 RSL3resis
shRNA-SCAF1-2+SCAF1resis
shRNA-SCAF1-2+Fer-1
% of cells with Oxidized C11-BODIPY
P<0.0001
n.s.
Ctrl
D
SNU-668 Erastinresis
SNU-484 RSL3resis
Cell viability (%)
P=0.0007
P=0.0024
P=0.0011
P=0.0060
Cisplatin (16 μM)
sh-SCAF1
SCAF1resis
Fer-1

**Supplementary Figure 13.** (A-D) SNU-668 Erastin$^{resis}$ cells or SNU-484 RSL3$^{resis}$ cells were transducted of lentiviruses encoding SCAF1 shRNA-1 or shRNA-2 or a scrambled shRNA and/or resistant SOX13 cDNA (SOX13$^{resis}$). (A) SCAF1 was determined by immunoblotting assay and normalized to β-actin (n=3 independent experiments). (B,C) Effect of knockdown of SCAF1 and SCAF1 re-expression on sensitivity of SNU-668 Erastin$^{resis}$ cells or SNU-484 RSL3$^{resis}$ cells to the ferroptosis inducers RSL3 or Erastin. Cells transfected with desired vector were treated with various concentrations of RSL3 or Erastin for 24 hours. (B) The heatmap shows altered cell viability. (C) SNU-484 RSL3$^{resis}$ cells transfected with desired vector were treated with Erastin (2 μM) or RSL3 (0.5 μM) for 24 hours. Lipid peroxidation was determined with a lipid peroxidation C11-BODIPY assay (n=3 independent experiments), and a representative flow cytometry histogram plot is presented. Data are presented as mean values ± SD. (D) Effect of SCAF1 downregulation on cisplatin sensitivity in the absence or presence of Fer-1 (1 μM) in Erastin$^{resis}$ SNU-668 cells or RSL3$^{resis}$ SNU-484 cells. The cells were treated with cisplatin (16 μM) for 24 hours. Cell viability was evaluated with a CellTiter-Glo luminescent cell viability assay (n=3 independent experiments). Data are presented as mean values ± SD. Statistical significance in (C,D) is determined by two-tailed unpaired *t*-test. Source data are provided as a Source Data file.

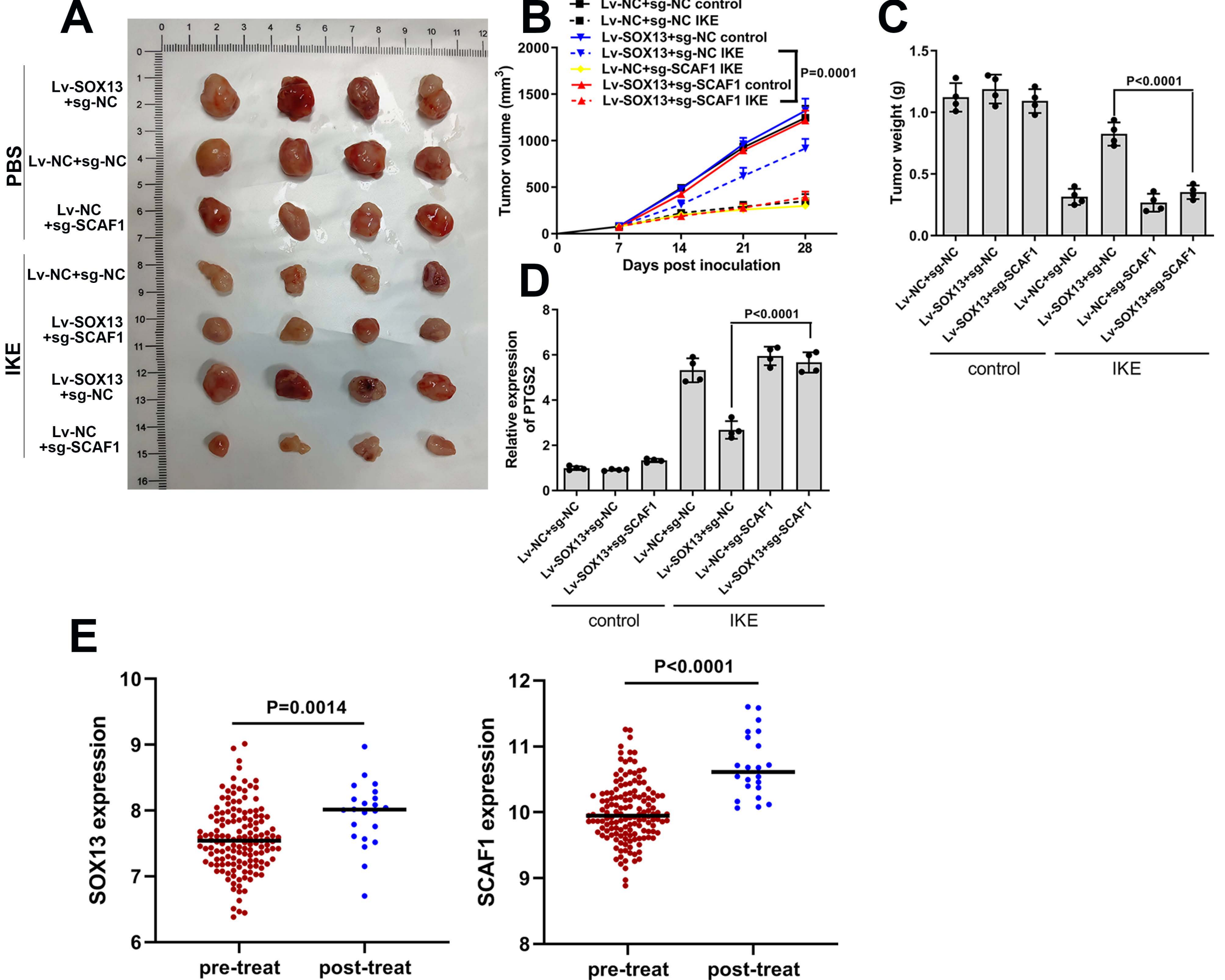

A
PBS
IKE
Lv-SOX13 +sg-NC
Lv-NC+sg-NC
Lv-NC +sg-SCAF1
Lv-NC+sg-NC
Lv-SOX13 +sg-SCAF1
Lv-SOX13 +sg-NC
Lv-NC +sg-SCAF1
B
Lv-NC+sg-NC control
Lv-NC+sg-NC IKE
Lv-SOX13+sg-NC control
Lv-SOX13+sg-NC IKE
Lv-NC+sg-SCAF1 IKE
Lv-SOX13+sg-SCAF1 control
Lv-SOX13+sg-SCAF1 IKE
P=0.0001
Tumor volume (mm3)
Days post inoculation
C
Tumor weight (g)
P<0.0001
Lv-NC+sg-NC
Lv-SOX13+sg-NC
Lv-SOX13+sg-SCAF1
Lv-NC+sg-NC
Lv-SOX13+sg-NC
Lv-NC+sg-SCAF1
Lv-SOX13+sg-SCAF1
control
IKE
D
Relative expression of PTGS2
P<0.0001
Lv-NC+sg-NC
Lv-SOX13+sg-NC
Lv-SOX13+sg-SCAF1
Lv-NC+sg-NC
Lv-SOX13+sg-NC
Lv-NC+sg-SCAF1
Lv-SOX13+sg-SCAF1
control
IKE
E
SOX13 expression
P=0.0014
pre-treat
post-treat
SCAF1 expression
P<0.0001
pre-treat
post-treat

**Supplementary Figure 14.** (A-D) SOX13-overexpressing CDX tumors (Lv-SOX13), SCAF1 KO-CDX tumors (sg-SCAF1) and control SNU-668-CDX tumors were treated with IKE 7 days after injection. Representative images of tumors formed (A), tumor growth curves (B), tumor weights (C), and PTGS2 expression (D) analysis are shown (n=4 mice per group). Data are presented as mean values ± SD. (E) SOX13 and SCAF1 expression levels were significantly increased after acquiring resistance to CF combination chemotherapy. Data are presented as mean values ± SD. Two-tailed unpaired t-test (B-D) or two-tailed paired t-test test (E). Source data are provided as a Source Data file.

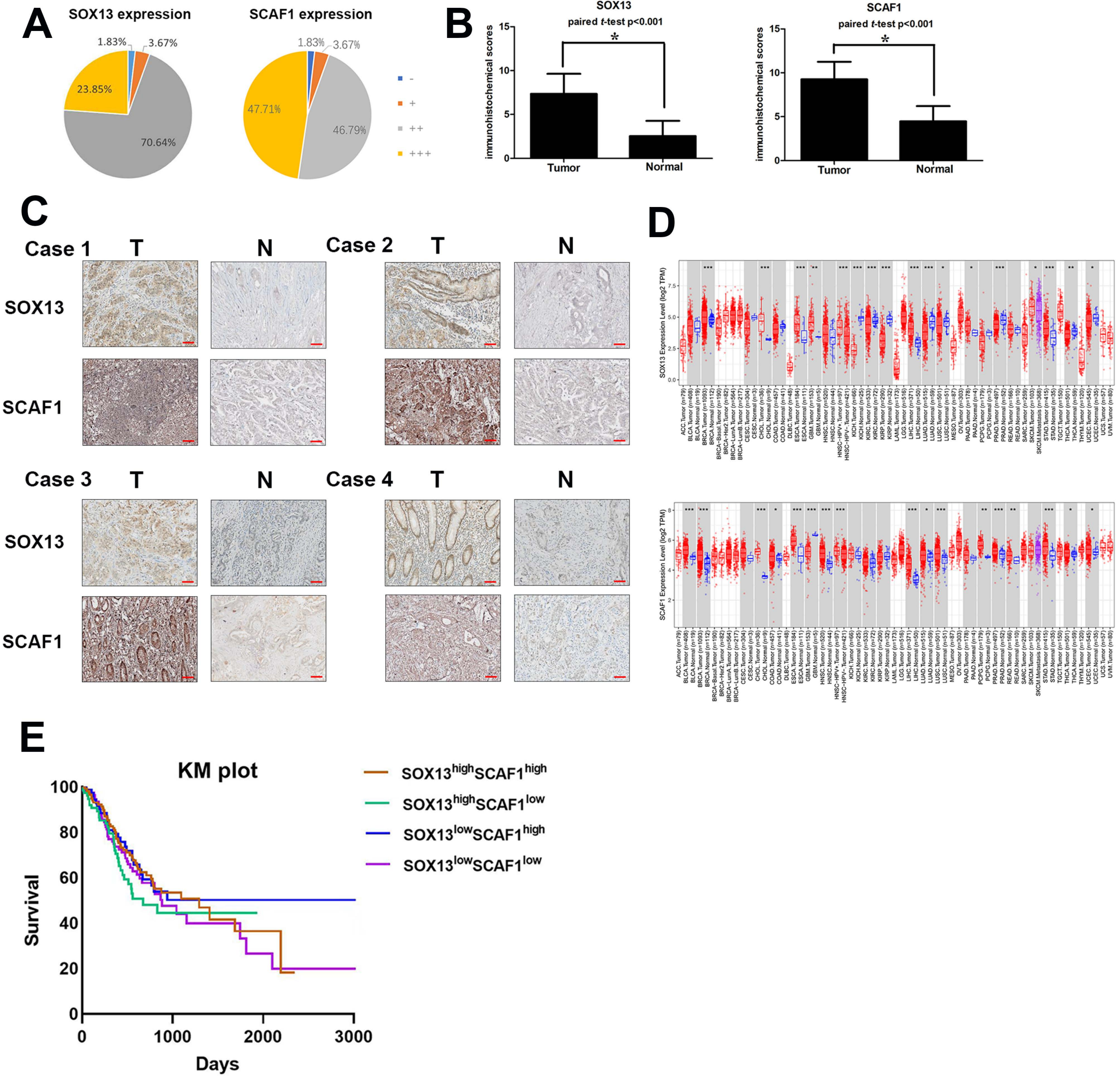
A
SOX13 expression
SCAF1 expression
1.83%
3.67%
23.85%
70.64%
47.71%
46.79%
-
+
++
+++
B
SOX13
SCAF1
paired t-test p<0.001
*
immunohistochemical scores
Tumor
Normal
C
Case 1
Case 2
Case 3
Case 4
T
N
SOX13
SCAF1
D
SOX13 Expression Level (log2 TPM)
SCAF1 Expression Level (log2 TPM)
E
KM plot
SOX13highSCAF1high
SOX13highSCAF1low
SOX13lowSCAF1high
SOX13lowSCAF1low
Survival
Days

**Supplementary Figure 15.** (A) Immunohistochemical staining of SOX13/SCAF1 in 109 GC samples and matched normal stomach tissues. The expression was scored as negative (-), low (+), intermediate (++), or high (+++). (B) SOX13/SCAF1 immunohistochemical scores in 109 GC tissues and matched normal stomach tissues (paired *t* test, $p<0.0001$). Data are presented as mean values ± SD. (C) Representative immunohistochemical staining images for SOX13/SCAF1 are presented. Scale bar: 50 μm. (D) Normalized expression of SOX13 and SCAF1 across different tumor types from TIMER data showing high overall expression in STAD. (E) Kaplan–Meier analysis of concurrent SOX13 and SCAF1 expression with overall survival in patients receiving surgery only according to data from TCGA-STAD. Two-tailed paired t-test (B) or two-sided log-rank test (E).

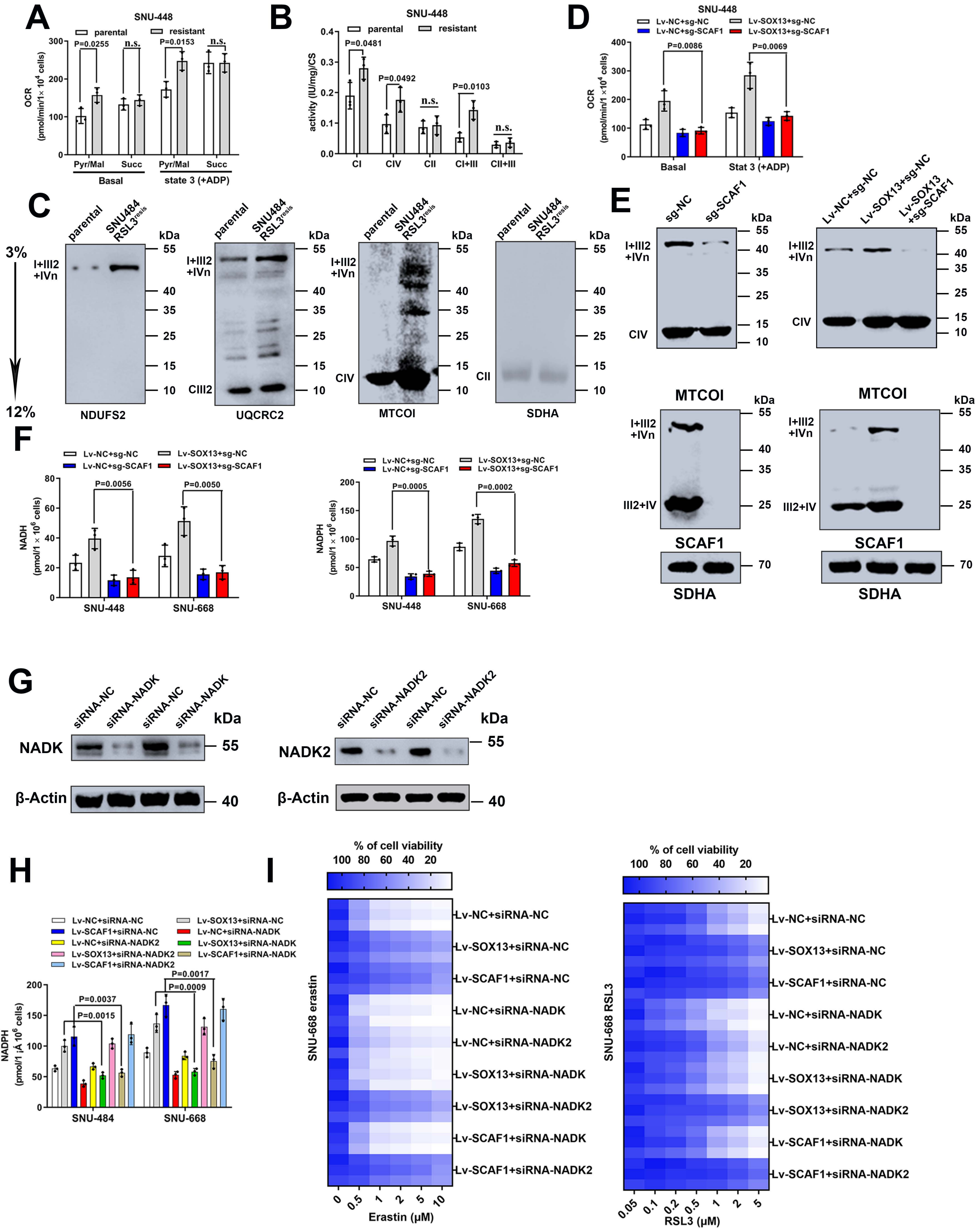

A
SNU-448
parental
resistant
P=0.0255
n.s.
P=0.0153
n.s.
OCR (pmol/min/1× 10^4 cells)
Pyr/Mal
Succ
Pyr/Mal
Succ
Basal
state 3 (+ADP)
B
SNU-448
parental
resistant
P=0.0481
P=0.0492
n.s.
P=0.0103
n.s.
activity (IU/mg)/CS
CI
CIV
CII
CI+III
CII+III
D
SNU-448
Lv-NC+sg-NC
Lv-SOX13+sg-NC
Lv-NC+sg-SCAF1
Lv-SOX13+sg-SCAF1
P=0.0086
P=0.0069
OCR (pmol/min/1 ×10^4 cells)
Basal
Stat 3 (+ADP)
C
3%
12%
parental
SNU484 RSL3resis
kDa
I+III2 +IVn
CIII2
CIV
CII
NDUFS2
UQCRC2
MTCOI
SDHA
E
sg-NC
sg-SCAF1
Lv-NC+sg-NC
Lv-SOX13+sg-NC
Lv-SOX13 +sg-SCAF1
I+III2 +IVn
CIV
MTCOI
III2+IV
SCAF1
SDHA
F
Lv-NC+sg-NC
Lv-SOX13+sg-NC
Lv-NC+sg-SCAF1
Lv-SOX13+sg-SCAF1
P=0.0056
P=0.0050
NADH (pmol/1 × 10^6 cells)
SNU-448
SNU-668
P=0.0005
P=0.0002
NADPH (pmol/1 × 10^6 cells)
G
siRNA-NC
siRNA-NADK
siRNA-NADK2
kDa
NADK
NADK2
β-Actin
H
Lv-NC+siRNA-NC
Lv-SOX13+siRNA-NC
Lv-SCAF1+siRNA-NC
Lv-NC+siRNA-NADK
Lv-NC+siRNA-NADK2
Lv-SOX13+siRNA-NADK
Lv-SOX13+siRNA-NADK2
Lv-SCAF1+siRNA-NADK
Lv-SCAF1+siRNA-NADK2
P=0.0037
P=0.0015
P=0.0017
P=0.0009
NADPH (pmol/1 ¡Á 10^6 cells)
SNU-484
SNU-668
I
% of cell viability
SNU-668 erastin
Erastin (μM)
SNU-668 RSL3
RSL3 (μM)
Lv-NC+siRNA-NC
Lv-SOX13+siRNA-NC
Lv-SCAF1+siRNA-NC
Lv-NC+siRNA-NADK
Lv-NC+siRNA-NADK2
Lv-SOX13+siRNA-NADK
Lv-SOX13+siRNA-NADK2
Lv-SCAF1+siRNA-NADK
Lv-SCAF1+siRNA-NADK2

**Supplementary Figure 16.** (A) Oxygen consumption analysis was performed in isolated mitochondria of parental and resistant SNU-484 cells using pyruvate-malate or succinate as specific substrates, respectively (n=3 independent experiments). Data are presented as mean values ± SD. (B) Mitochondrial enzymatic activities of CI, CIV, CII, CI+III, and CII+III normalized to citrate synthase (CS) levels in parental and resistant SNU-484 cells (n=3 independent experiments). Data are presented as mean values ± SD. (C) Western blot analysis of the indicated proteins, including CI, CIII, CIV, and CII, after blue native PAGE (BN-PAGE) of digitonin-solubilized mitochondria from parental and resistant SNU-484 cells (n=3 independent experiments). (D) Oxygen consumption rates of isolated mitochondria from parental SNU-484 cells with SOX13 overexpression (Lv-SOX13) alone, SCAF1 KO (sg-SCAF1) alone or the two in combination (n=3 independent experiments). Data are presented as mean values ± SD. (E) SC levels in parental SNU-484 cells with SOX13 overexpression (Lv-SOX13) alone, SCAF1 KO (sg-SCAF1) alone or the two in combination (n=3 independent experiments). (F) The increased NADH/NADPH production in parental cells induced by SOX13 overexpression was attenuated with SCAF1 silencing (n=3 independent experiments). Data are presented as mean values ± SD. (G) NADK and NADK2 protein expression levels were measured by Western blot analysis (n=3 independent experiments). β-Actin served as a loading control. (H) The cellular NADPH level was significantly decreased with silencing of the cytosolic enzyme NADK; however, downregulation of the mitochondrial enzyme NADK2 had no such effects (n=3 independent experiments). Data are presented as mean values ±

SD. (I) The survival benefit of SOX13/SCAF1-overexpressing parental SNU-668 cells was largely eliminated with NADK silencing. The cells were exposed to RSL3 (0.5 μM) or Erastin (2 μM) for 24 hours. Statistical significance in (A,B,D,F,H) is determined by two-tailed unpaired *t*-test. Source data are provided as a Source Data file.

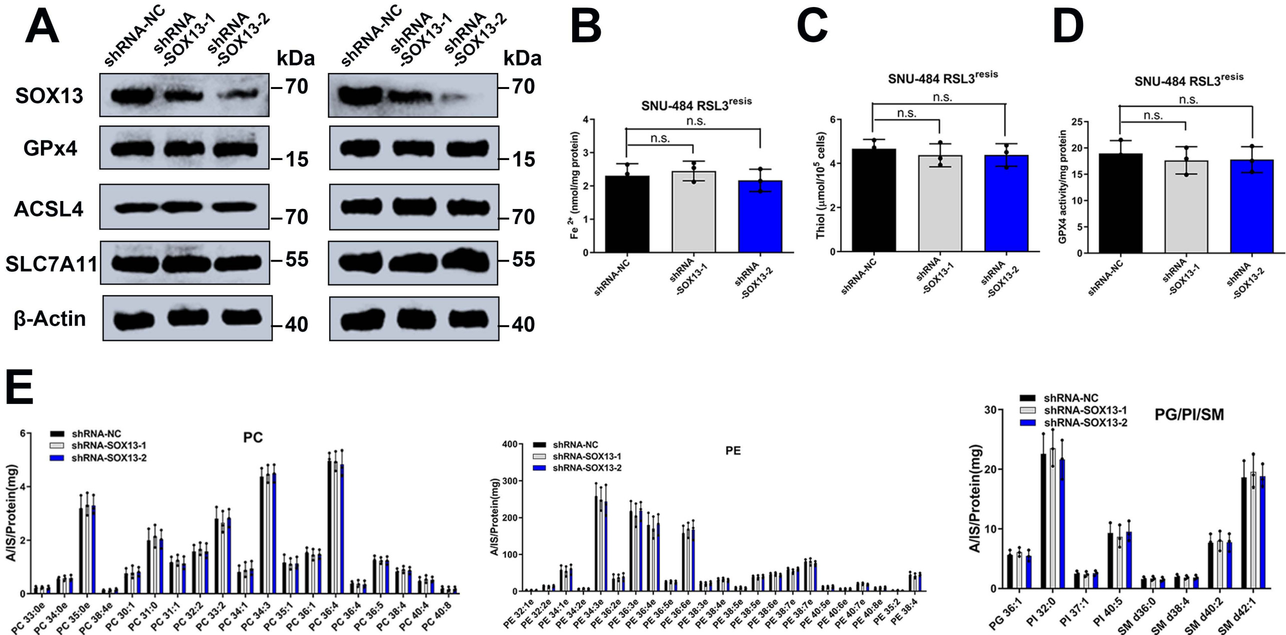
A
shRNA-NC
shRNA -SOX13-1
shRNA -SOX13-2
kDa
SOX13
GPx4
ACSL4
SLC7A11
β-Actin
70
15
70
55
40
B
SNU-484 RSL3resis
Fe 2+ (nmol/mg protein)
n.s.
C
SNU-484 RSL3resis
Thiol (μmol/10^5 cells)
D
SNU-484 RSL3resis
GPX4 activity/mg protein
E
shRNA-NC
shRNA-SOX13-1
shRNA-SOX13-2
PC
PE
PG/PI/SM
A/IS/Protein(mg)
PC 33:0e
PC 34:0e
PC 35:0e
PC 38:4e
PC 30:1
PC 31:0
PC 31:1
PC 32:2
PC 33:2
PC 34:1
PC 34:3
PC 35:1
PC 36:1
PC 36:4
PC 36:4
PC 36:5
PC 38:4
PC 40:4
PC 40:8
PE 32:1e
PE 32:2e
PE 34:1e
PE 34:2e
PE 34:3e
PE 36:2e
PE 36:3e
PE 36:4e
PE 36:5e
PE 36:6e
PE 38:3e
PE 38:4e
PE 38:5e
PE 38:5e
PE 38:6e
PE 38:7e
PE 38:7e
PE 40:5e
PE 40:6e
PE 40:7e
PE 40:8e
PE 35:2
PE 38:4
PG 36:1
PI 32:0
PI 37:1
PI 40:5
SM d36:0
SM d38:4
SM d40:2
SM d42:1

**Supplementary Figure 17.** (A-E) Effect of SOX13 knockdown on the expression of GPX4, SLC7A11, and ACSL4 (A), cellular divalent iron levels (B), system $Xc^-$ (C) and GPX4 activity (D) or cellular phospholipid composition (E). (A) GPX4, SLC7A11, and ACSL4 protein expression levels were measured by Western blot analysis (n=3 independent experiments). β-Actin served as a loading control. Cellular divalent iron levels (B), system $Xc^-$ (C) and GPX4 activity (D) were determined as described in the Materials and methods in SNU-484 $RSL3^{resis}$ cells (n=3 independent experiments). Data are presented as mean values ± SD. (E) Lipidomic profile of SNU-484 $RSL3^{resis}$ (control or SOX13 downregulated) cells. The data represents the mean value of the ratio of the area of analyte (A)/internal standard (IS)/protein (mg). Each group has three replicates, and results were representative of three independent experiments. Data are presented as mean values ± SD. Statistical significance in (B-E) is determined by two-tailed unpaired *t*-test. Source data are provided as a Source Data file.

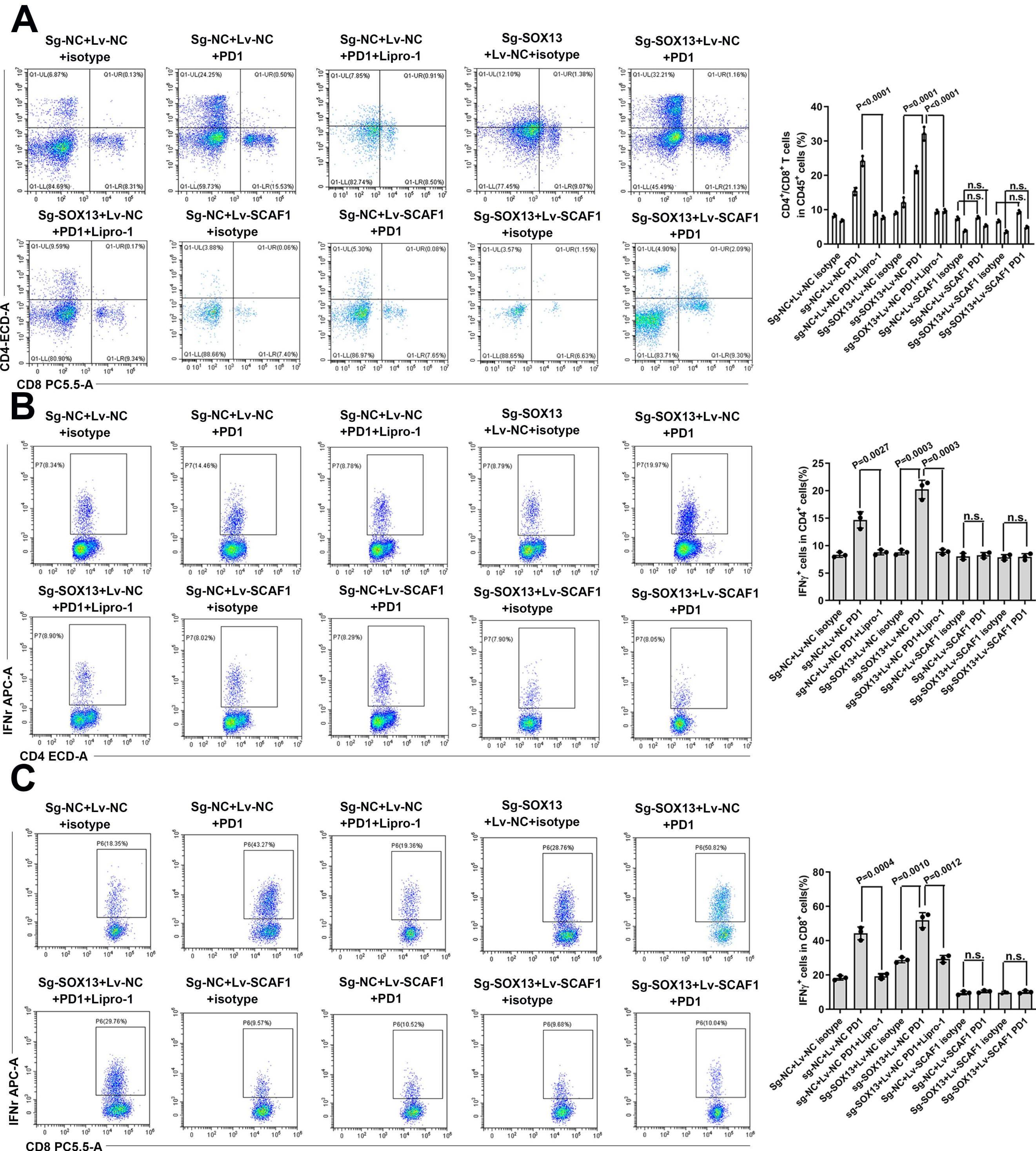
A
Sg-NC+Lv-NC +isotype
Sg-NC+Lv-NC +PD1
Sg-NC+Lv-NC +PD1+Lipro-1
Sg-SOX13 +Lv-NC+isotype
Sg-SOX13+Lv-NC +PD1
Sg-SOX13+Lv-NC +PD1+Lipro-1
Sg-NC+Lv-SCAF1 +isotype
Sg-NC+Lv-SCAF1 +PD1
Sg-SOX13+Lv-SCAF1 +isotype
Sg-SOX13+Lv-SCAF1 +PD1
CD4-ECD-A
CD8 PC5.5-A
CD4+/CD8+ T cells in CD45+ cells (%)
P<0.0001
P=0.0001
P<0.0001
n.s.
B
IFNr APC-A
CD4 ECD-A
IFNγ+ cells in CD4+ cells(%)
P=0.0027
P=0.0003
P=0.0003
n.s.
C
IFNr APC-A
CD8 PC5.5-A
IFNγ+ cells in CD8+ cells(%)
P=0.0004
P=0.0010
P=0.0012
n.s.
Sg-NC+Lv-NC isotype
sg-NC+Lv-NC PD1
sg-NC+Lv-NC PD1+Lipro-1
Sg-SOX13+Lv-NC isotype
sg-SOX13+Lv-NC PD1
sg-SOX13+Lv-NC PD1+Lipro-1
Sg-NC+Lv-SCAF1 isotype
Sg-NC+Lv-SCAF1 PD1
Sg-SOX13+Lv-SCAF1 isotype
Sg-SOX13+Lv-SCAF1 PD1

**Supplementary Figure 18.** (A-C) Representative flow cytometry analysis and quantification of the indicated $CD8^+$ and $CD4^+$ TILs in YTH16 (m)-derived xenografts with the treatment described above (n=3 independent experiments). Data are presented as mean values ± SD. Statistical significance in (A-C) is determined by two-tailed unpaired *t*-test. Source data are provided as a Source Data file.

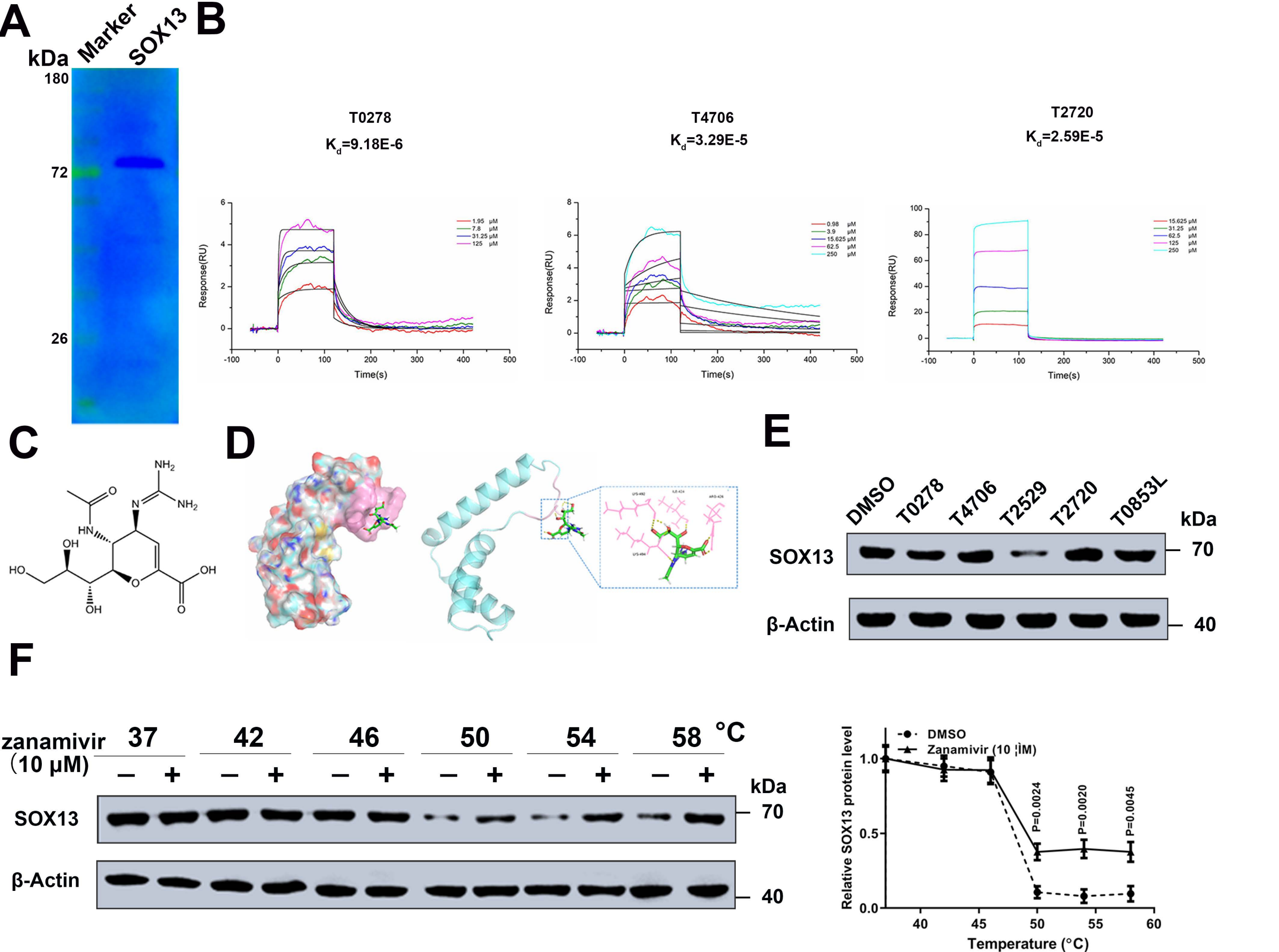
A
B
C
D
E
F
kDa
Marker
SOX13
180
72
26
T0278
$K_d$=9.18E-6
T4706
$K_d$=3.29E-5
T2720
$K_d$=2.59E-5
Response(RU)
Time(s)
DMSO
T0278
T4706
T2529
T2720
T0853L
SOX13
β-Actin
70
40
zanamivir
(10 μM)
37
42
46
50
54
58
°C
Relative SOX13 protein level
Temperature (°C)
DMSO
Zanamivir (10 μM)
P=0.0024
P=0.0020
P=0.0045

**Supplementary Figure 19.** (A) Coomassie blue staining showing the purity of recombinant human SOX13 protein. (B) SPR analysis results showing the binding of the SOX13 protein to several other small molecules (T0278, T4706 and T2720). (C) Chemical structures of zanamivir. (D) Computational molecular docking analysis to investigate the interaction of zanamivir binding to SOX13. The green molecule represents zanamivir. (E) Western blot analysis of the expression of SOX13 in Erastin$^{resis}$ SNU-668 cells treated with different compounds (10 μM) for 36 h (n=3 independent experiments). (F) Melt curves of SOX13 protein in CETSA in Erastin$^{resis}$ SNU-668 cells treated with zanamivir or vehicle for 1 h. The graph shows the quantification of SOX13 protein versus temperature points based on Western blot analyses (n=3 independent experiments). Data are presented as mean values ± SD. Statistical significance in (F) is determined by two-tailed unpaired *t*-test. Source data are provided as a Source Data file.

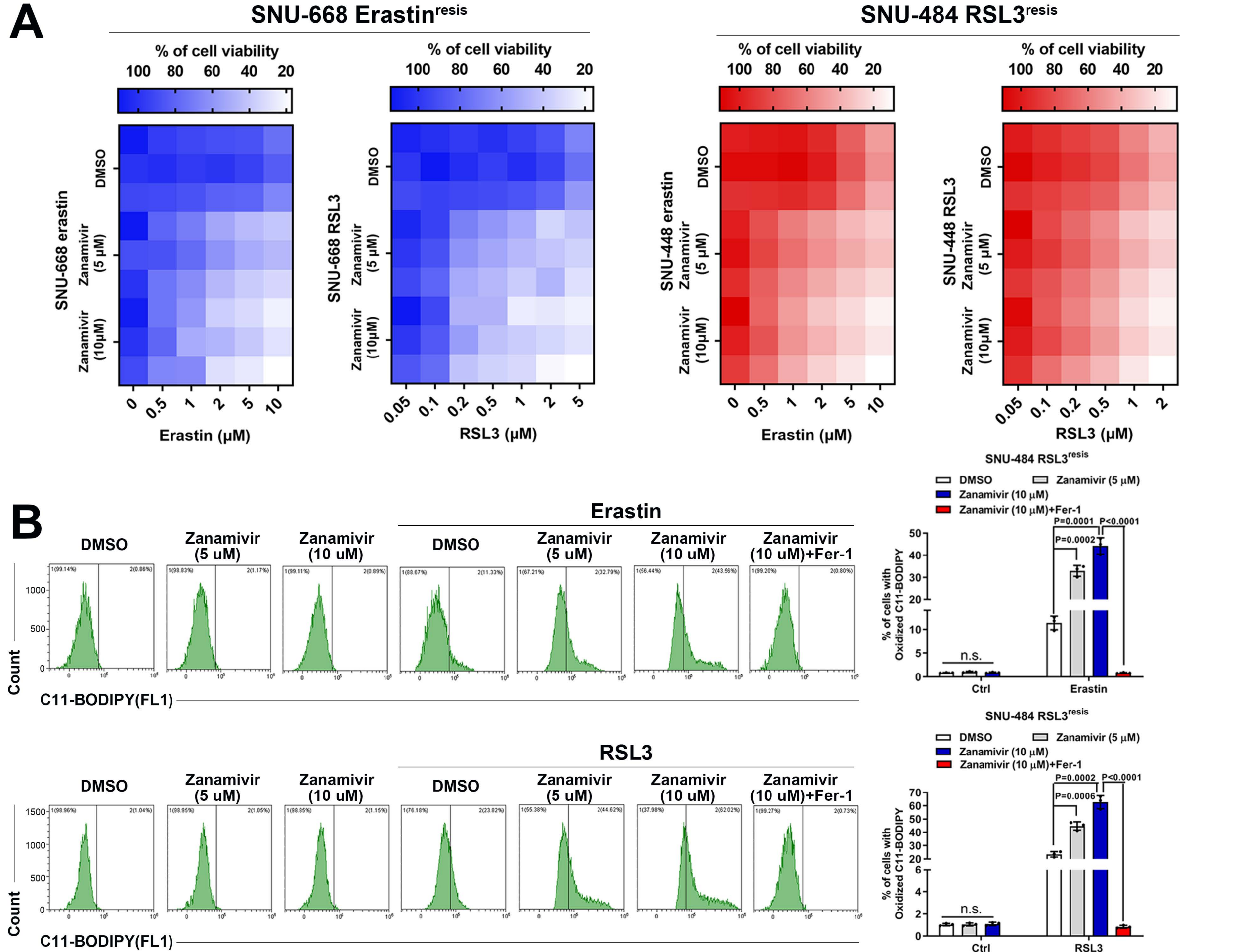
A
SNU-668 Erastin^resis
SNU-484 RSL3^resis
% of cell viability
100 80 60 40 20
SNU-668 erastin
SNU-668 RSL3
SNU-448 erastin
SNU-448 RSL3
DMSO
Zanamivir (5 μM)
Zanamivir (10μM)
0 0.5 1 2 5 10
Erastin (μM)
0.05 0.1 0.2 0.5 1 2 5
RSL3 (μM)
B
Erastin
RSL3
DMSO
Zanamivir (5 uM)
Zanamivir (10 uM)
Zanamivir (10 uM)+Fer-1
Count
C11-BODIPY(FL1)
SNU-484 RSL3^resis
DMSO
Zanamivir (5 μM)
Zanamivir (10 μM)
Zanamivir (10 μM)+Fer-1
% of cells with Oxidized C11-BODIPY
n.s.
P=0.0001
P<0.0001
P=0.0002
P=0.0006
Ctrl
Erastin
RSL3

**Supplementary Figure 20.** (A) The Erastin$^{resis}$ SNU-668 cells or RSL3-resistant SNU-484 cells were pretreated with zanamivir (0 μM, 5 μM, 10 μM) for 12 h and then exposed to various concentrations of RSL3 or Erastin for 24 hours. The heatmap shows altered cell viability. (B) The RSL3$^{resis}$ SNU-484 cells cells were pretreated with zanamivir (0 μM, 5 μM, 10 μM) for 12h and then exposed to Erastin (2 μM) or RSL3 (0.5 μM) for 24 hours. Lipid peroxidation was determined using a lipid peroxidation C11-BODIPY assay (n=3 independent experiments), and representative flow cytometry histogram plot is presented. Data are presented as mean values ± SD. Statistical significance in (B) is determined by two-tailed unpaired *t*-test. Source data are provided as a Source Data file.

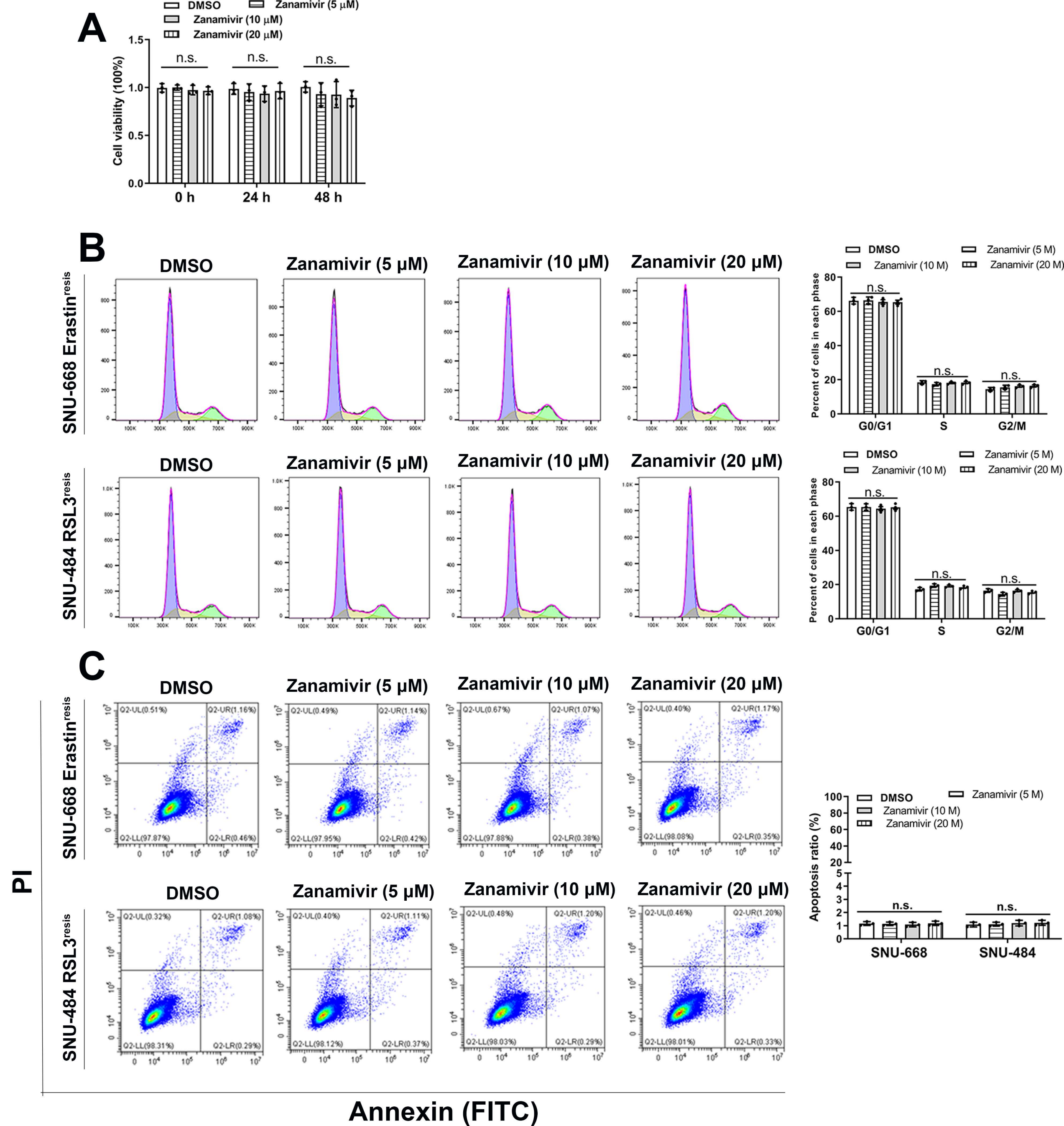

A
DMSO
Zanamivir (5 μM)
Zanamivir (10 μM)
Zanamivir (20 μM)
Cell viability (100%)
n.s.
0 h
24 h
48 h
B
SNU-668 Erastin^resis
SNU-484 RSL3^resis
DMSO
Zanamivir (5 μM)
Zanamivir (10 μM)
Zanamivir (20 μM)
Percent of cells in each phase
G0/G1
S
G2/M
C
Q2-UL(0.51%) Q2-UR(1.16%) Q2-LL(97.87%) Q2-LR(0.46%)
Q2-UL(0.49%) Q2-UR(1.14%) Q2-LL(97.95%) Q2-LR(0.42%)
Q2-UL(0.67%) Q2-UR(1.07%) Q2-LL(97.88%) Q2-LR(0.38%)
Q2-UL(0.40%) Q2-UR(1.17%) Q2-LL(98.08%) Q2-LR(0.35%)
Q2-UL(0.32%) Q2-UR(1.08%) Q2-LL(98.31%) Q2-LR(0.29%)
Q2-UL(0.40%) Q2-UR(1.11%) Q2-LL(98.12%) Q2-LR(0.37%)
Q2-UL(0.48%) Q2-UR(1.20%) Q2-LL(98.03%) Q2-LR(0.29%)
Q2-UL(0.46%) Q2-UR(1.20%) Q2-LL(98.01%) Q2-LR(0.33%)
Apoptosis ratio (%)
SNU-668
SNU-484
PI
Annexin (FITC)

**Supplementary Figure 21.** (A-C) The Erastin$^{resis}$ SNU-668 cells or RSL3$^{resis}$ SNU-484 cells were pretreated with zanamivir (0 μM, 5 μM, 10 μM, 20 μM) for 48 h (A) or 36 h (B-C). (A) Relative viability was determined in Erastin$^{resis}$ SNU-668 cells (n=3 independent experiments). Data are presented as mean values ± SD. (B) Cell cycle analysis determined the relative cell numbers in each cell-cycle phase with propidium iodide staining (n=3 independent experiments). Data are presented as mean values ± SD. (C) Annexin V/PI staining and flow cytometry analysis assessing apoptosis (n=3 independent experiments). Data are presented as mean values ± SD. Statistical significance in (A-C) is determined by two-tailed unpaired *t*-test. Source data are provided as a Source Data file.

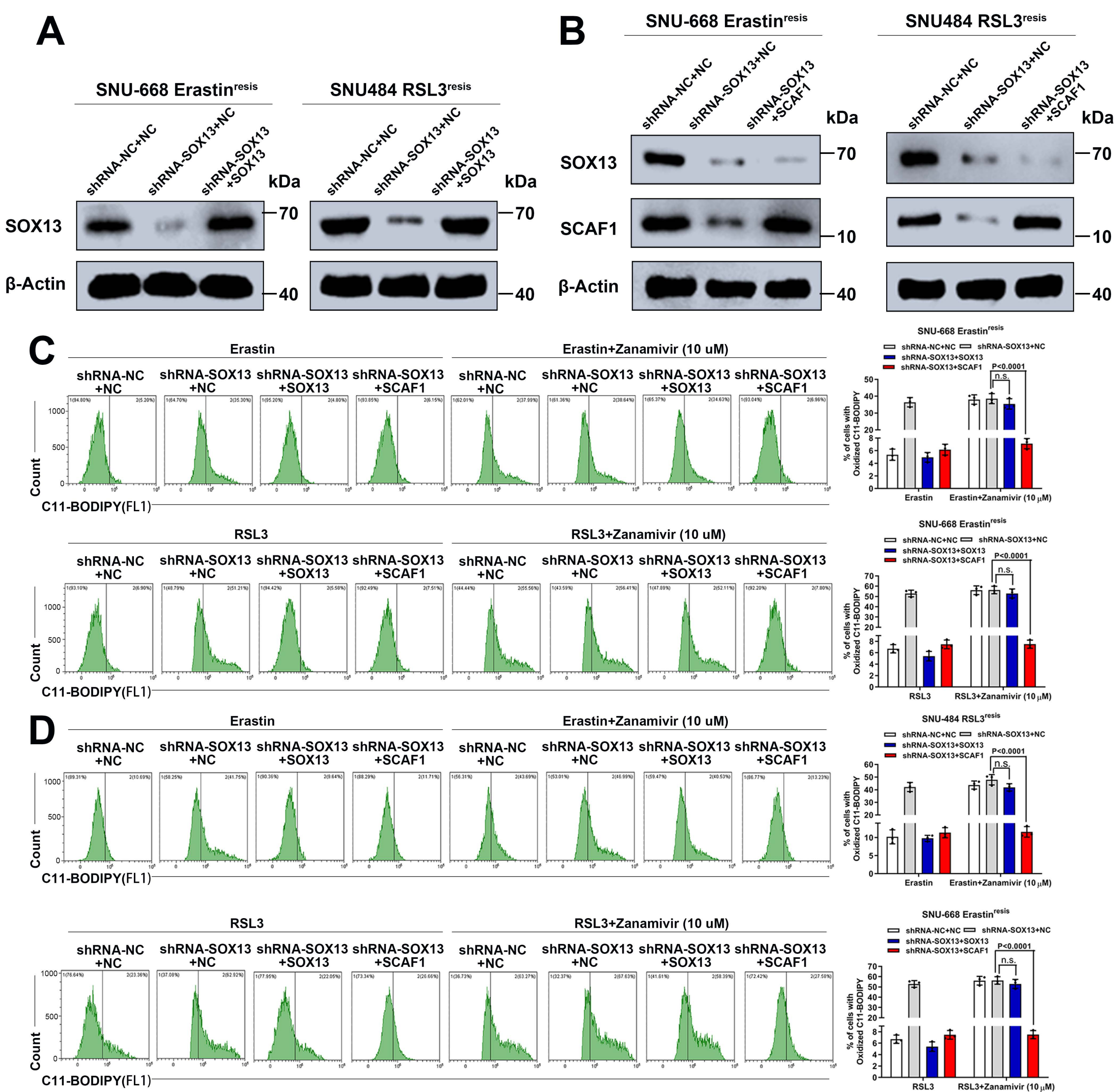
A
SNU-668 Erastin resis
SNU484 RSL3 resis
shRNA-NC+NC
shRNA-SOX13+NC
shRNA-SOX13 +SOX13
kDa
SOX13
β-Actin
70
40
B
SNU-668 Erastin resis
SNU484 RSL3 resis
shRNA-SOX13 +SCAF1
SCAF1
10
C
Erastin
Erastin+Zanamivir (10 uM)
shRNA-NC +NC
shRNA-SOX13 +NC
shRNA-SOX13 +SOX13
shRNA-SOX13 +SCAF1
Count
C11-BODIPY(FL1)
SNU-668 Erastin resis
shRNA-SOX13+SOX13
shRNA-SOX13+SCAF1
P<0.0001
n.s.
% of cells with Oxidized C11-BODIPY
Erastin+Zanamivir (10 μM)
RSL3
RSL3+Zanamivir (10 uM)
RSL3+Zanamivir (10 μM)
D
SNU-484 RSL3 resis

**Supplementary Figure 22.** (A,B) Wild-type SOX13 (A) or SCAF1 (B) were re-overexpressed in SOX13-deficient FINs resistant cells. SOX13, and SCAF1 protein expression levels were measured by Western blot analysis (n=3 independent experiments). (C,D) Wild-type SOX13 or SCAF1 were re-overexpressed in SOX13-deficient Erastin$^{resis}$ SNU-668 cells (C) or RSL3$^{resis}$ SNU-484 cells (D). Cells were pretreated with zanamivir (0 μM, 10 μM) for 12h and exposed to Erastin (2 μM) or RSL3 (0.5 μM) for 24 hours. The sensitivity of cells to FINs was evaluated with a lipid peroxidation C11-BODIPY assay (n=3 independent experiments). Data are presented as mean values ± SD. Statistical significance in (C,D) is determined by two-tailed unpaired *t*-test. Source data are provided as a Source Supplementary Figure 22. (A,B) Wild-type SOX13 (A) or SCAF1 (B) were re-overexpressed in SOX13-deficient FINs resistant cells. SOX13, and SCAF1 protein expression levels were measured by Western blot analysis (n=3 independent experiments). (C,D) Wild-type SOX13 or SCAF1 were re-overexpressed in SOX13-deficient Erastinresis SNU-668 cells (C) or RSL3resis SNU-484 cells (D). Cells were pretreated with zanamivir (0 μM, 10 μM) for 12h and exposed to Erastin (2 μM) or RSL3 (0.5 μM) for 24 hours. The sensitivity of cells to FINs was evaluated with a lipid peroxidation C11-BODIPY assay (n=3 independent experiments). Data are presented as mean values ± SD. Statistical significance in (C,D) is determined by two-tailed unpaired t-test. Source data are provided as a Source Data file.

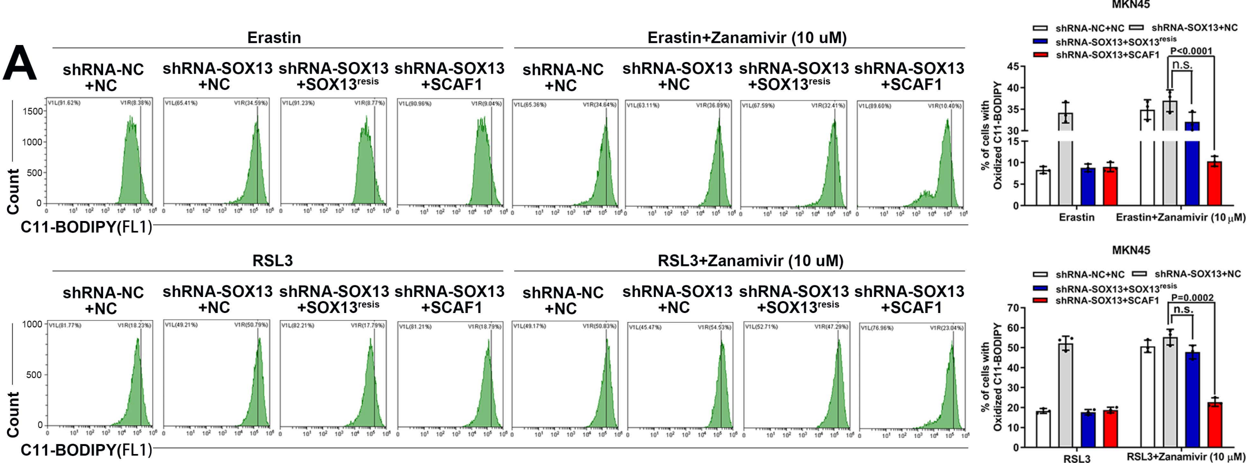
A
Erastin
Erastin+Zanamivir (10 uM)
shRNA-NC +NC
shRNA-SOX13 +NC
shRNA-SOX13 +SOX13resis
shRNA-SOX13 +SCAF1
Count
C11-BODIPY(FL1)
MKN45
shRNA-NC+NC
shRNA-SOX13+NC
shRNA-SOX13+SOX13resis
shRNA-SOX13+SCAF1
P<0.0001
n.s.
% of cells with Oxidized C11-BODIPY
Erastin
Erastin+Zanamivir (10 μM)
RSL3
RSL3+Zanamivir (10 uM)
MKN45
P=0.0002
n.s.
RSL3
RSL3+Zanamivir (10 μM)

**Supplementary Figure 23.** Wild-type SOX13 or SCAF1 were re-overexpressed in SOX13-deficient MKN45 cells. Cells were pretreated with zanamivir (0 μM, 10 μM) for 12h and exposed to Erastin (2 μM) or RSL3 (0.5 μM) for 24 hours. The sensitivity of cells to FINs was evaluated with a lipid peroxidation C11-BODIPY assay (n=3 independent experiments). Data are presented as mean values ± SD. Statistical significance in (A) is determined by two-tailed unpaired *t*-test. Source data are provided as a Source Data file.

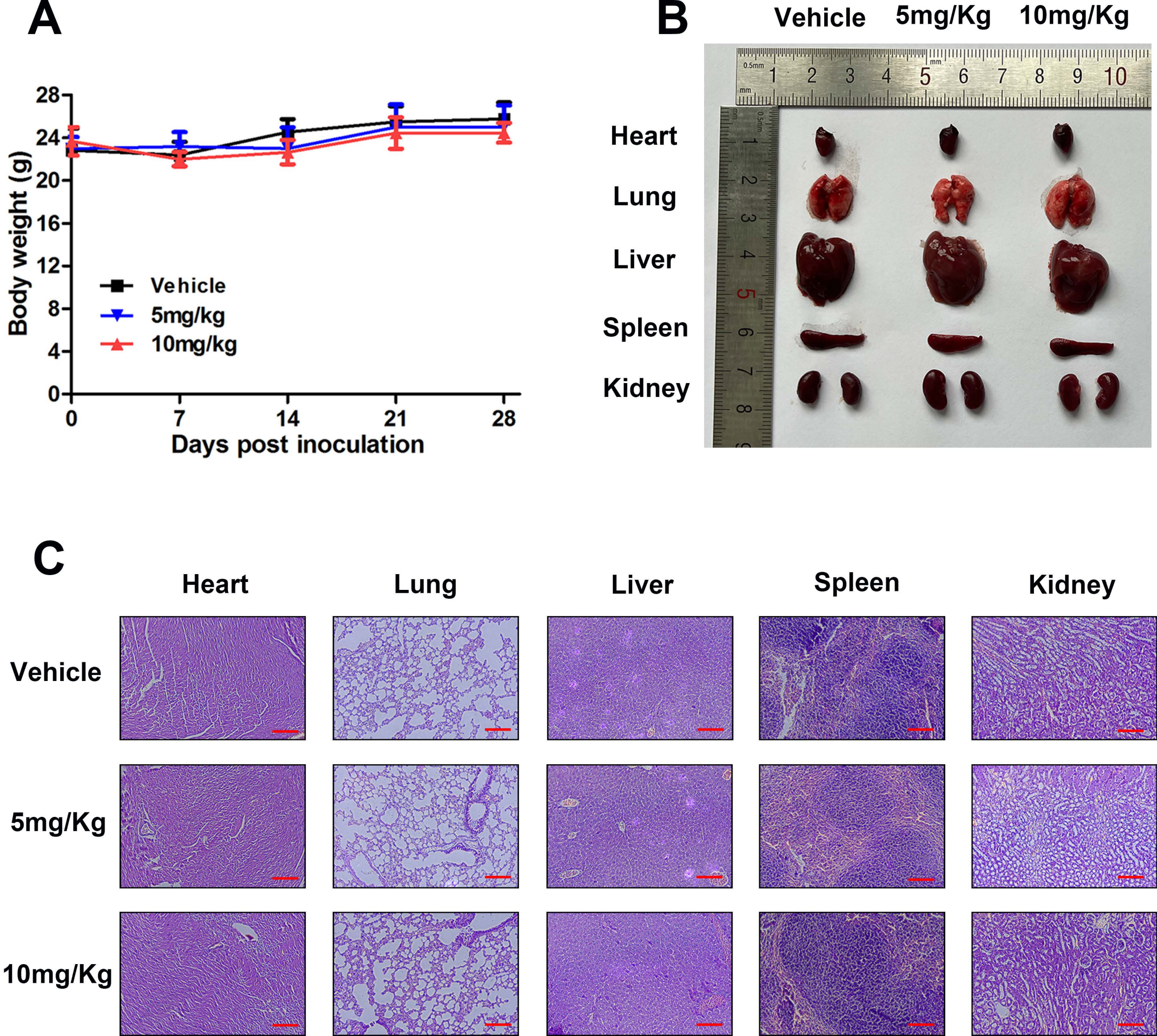
A
Body weight (g)
28
24
20
16
12
8
4
0
0
7
14
21
28
Days post inoculation
Vehicle
5mg/kg
10mg/kg
B
Vehicle
5mg/Kg
10mg/Kg
Heart
Lung
Liver
Spleen
Kidney
C
Heart
Lung
Liver
Spleen
Kidney
Vehicle
5mg/Kg
10mg/Kg

**Supplementary Figure 24. (**A) Body weights of mice in 28 days of treatment with vehicle or zanamivir (5 mg/kg and 10 mg/kg). (B) The size and appearance of heart, liver, spleen, lung and kidney of each group in Erastin$^{resis}$ SNU-668 xenograft model. (C) HE staining of heart, liver, spleen, lung and kidney of each group. Scale bars: 200 μm (100× magnification).

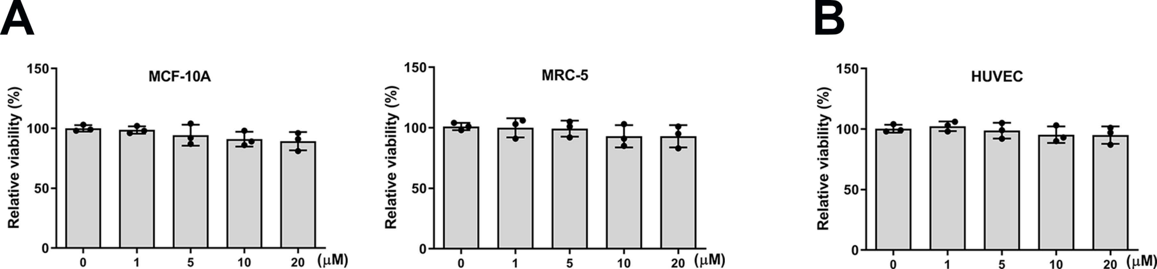

A
B
MCF-10A
MRC-5
HUVEC
Relative viability (%)
150
100
50
0
0
1
5
10
20
(μM)

**Supplementary Figure 25.** Toxicity of zanamivir in non-tumorigenic epithelial and endothelial cells. (A) Zanamivir has little effect on the viability of non-tumorigenic mammary and pulmonary epithelial cells, MCF-10A and MRC-5 (n=3 independent experiments). (B) Zanamivir has no effect on the viability of human umbilical vein endothelial cells HUVEC (n=3 independent experiments). Data are presented as mean values ± SD. Statistical significance in (A,B) is determined by two-tailed unpaired *t*-test. Source data are provided as a Source Data file.

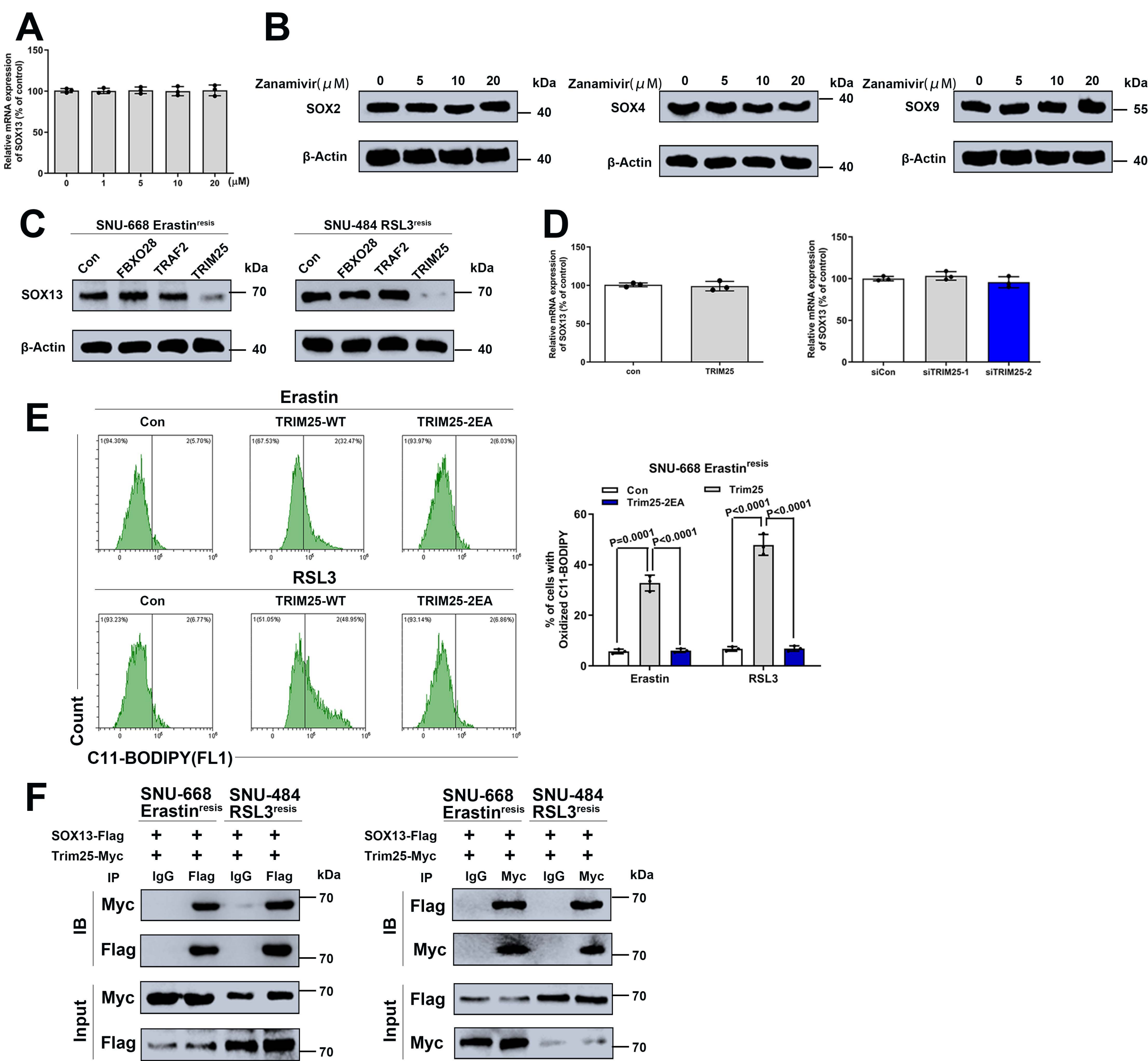
A
Relative mRNA expression of SOX13 (% of control)
0
1
5
10
20 (μM)
B
Zanamivir(μM) 0 5 10 20 kDa
SOX2
β-Actin
SOX4
SOX9
40
55
C
SNU-668 Erastin^resis
SNU-484 RSL3^resis
Con FBXO28 TRAF2 TRIM25 kDa
SOX13
β-Actin
70
40
D
con
TRIM25
siCon
siTRIM25-1
siTRIM25-2
E
Erastin
RSL3
Con
TRIM25-WT
TRIM25-2EA
1(94.30%) 2(5.70%)
1(67.53%) 2(32.47%)
1(93.97%) 2(6.03%)
1(93.23%) 2(6.77%)
1(51.05%) 2(48.95%)
1(93.14%) 2(6.86%)
Count
C11-BODIPY(FL1)
SNU-668 Erastin^resis
Con
Trim25
Trim25-2EA
% of cells with Oxidized C11-BODIPY
P=0.0001
P<0.0001
Erastin
RSL3
F
SNU-668 Erastin^resis
SNU-484 RSL3^resis
SOX13-Flag + + + +
Trim25-Myc + + + +
IP IgG Flag IgG Flag kDa
IP IgG Myc IgG Myc kDa
IB
Input
Myc
Flag
70

**Supplementary Figure 26.** (A) qRT-PCR results showing the mRNA expression of SOX13 in Erastin$^{resis}$ SNU-668 cells treated with zanamivir (0 μM, 5 μM, 10 μM, and 20 μM) for 36 h (n=3 independent experiments). Data are presented as mean values ± SD. (B) The Erastin$^{resis}$ SNU-668 cells were pretreated with zanamivir (0 μM, 5 μM, 10 μM, 20 μM) for 36 h. SOX2, SOX4, and SOX9 protein expression levels were measured by Western blot analysis and normalized to β-Actin (n=3 independent experiments). (C) Western blot analysis of SOX13 expression in Erastin$^{resis}$ SNU-668 cells or RSL3$^{resis}$ SNU-484 cells overexpressing TRIM25, FBXO28 or TRAF2 (n=3 independent experiments). (D) qRT-PCR results showing the expression level of SOX13 in TRIM25-overexpressing and TRIM25-knockdown Erastin$^{resis}$ SNU-668 cells (n=3 independent experiments). Data are presented as mean values ± SD. (E) TRIM25-WT, TRIM25-2EA or vector control were overexpressed in Erastin$^{resis}$ SNU-668 cells. The cells were treated with zanamivir (10 μM) for 12h before exposed to Erastin (2 μM) or RSL3 (0.5 μM) for 24 hours. Lipid peroxidation was determined utilizing a lipid peroxidation C11-BODIPY assay (n=3 independent experiments), and representative flow cytometry histogram plot is presented. Data are presented as mean values ± SD. (F) Immunoprecipitation results showing the interaction between SOX13 and TRIM25 (n=3 independent experiments). Statistical significance in (A,D,E) is determined by two-tailed unpaired *t*-test. Source data are provided as a Source Data file.
