# Supplementary material for: Targeting SOX13 inhibits assembly of respiratory chain supercomplexes to overcome ferroptosis resistance in gastric cancer
Source: Nat Commun. 2024 May 20;15:4296. doi: 10.1038/s41467-024-48307-z (PMC11106335; doi:10.1038/s41467-024-48307-z)
Supplement: Supplementary file 2 — Reporting Summary [file 41467_2024_48307_MOESM2_ESM.pdf]

nature portfolio

Corresponding author(s): Mingzhe Ma

Last updated by author(s): Mar 13, 2024

Reporting Summary

Nature Portfolio wishes to improve the reproducibility of the work that we publish. This form provides structure for consistency and transparency in reporting. For further information on Nature Portfolio policies, see our Editorial Policies and the Editorial Policy Checklist.

Statistics

For all statistical analyses, confirm that the following items are present in the figure legend, table legend, main text, or Methods section.

n/a	Confirmed	
☐	☒	The exact sample size (*n*) for each experimental group/condition, given as a discrete number and unit of measurement
☐	☒	A statement on whether measurements were taken from distinct samples or whether the same sample was measured repeatedly
☐	☒	The statistical test(s) used AND whether they are one- or two-sided<br>*Only common tests should be described solely by name; describe more complex techniques in the Methods section.*
☒	☐	A description of all covariates tested
☐	☒	A description of any assumptions or corrections, such as tests of normality and adjustment for multiple comparisons
☐	☒	A full description of the statistical parameters including central tendency (e.g. means) or other basic estimates (e.g. regression coefficient) AND variation (e.g. standard deviation) or associated estimates of uncertainty (e.g. confidence intervals)
☐	☒	For null hypothesis testing, the test statistic (e.g. *F*, *t*, *r*) with confidence intervals, effect sizes, degrees of freedom and *P* value noted<br>*Give P values as exact values whenever suitable.*
☒	☐	For Bayesian analysis, information on the choice of priors and Markov chain Monte Carlo settings
☒	☐	For hierarchical and complex designs, identification of the appropriate level for tests and full reporting of outcomes
☒	☐	Estimates of effect sizes (e.g. Cohen's *d*, Pearson's *r*), indicating how they were calculated

Our web collection on statistics for biologists contains articles on many of the points above.

Software and code

Policy information about availability of computer code

Data collection	See methods "Flow cytometry and cell sorting", "RNA isolation and qRT-PCR","Immunoblotting analysis","CHIP-Seq","Cell viability and cytotoxicity", "Cell apoptosis assay", "Cell cycle analysis","In vivo xenograft assays", Specific softwares include CytExpert+2.0; Analysis software: FlowJo_v10.6.2 and Modfit 5.0, MACS v2.1.0
Data analysis	Data are presented as the mean ± standard deviation of three or four biologically independent repeats. The data were analyzed with GraphPad Prism 8.0 (GraphPad Software, La Jolla, CA). Error bars are not visible in cases where the error is relatively smaller than the symbol. Statistical differences between pairs of groups were analyzed by independent two-tailed Student’s t-test. Survival differences were assessed by Kaplan–Meier methods, and statistical significance was determined by the log-rank test. The correlation between SOX13/SCAF1 coexpression scores and tumor regression grade was estimated with the nonparametric Mann-Whitney Wilcoxon test. The correlation between SOX13 and SCAF1 expression levels was determined using Pearson correlation analysis. *, $p < 0.05$; ns, not significant.

For manuscripts utilizing custom algorithms or software that are central to the research but not yet described in published literature, software must be made available to editors and reviewers. We strongly encourage code deposition in a community repository (e.g. GitHub). See the Nature Portfolio guidelines for submitting code & software for further information.

Data

Policy information about availability of data

All manuscripts must include a data availability statement. This statement should provide the following information, where applicable:

- Accession codes, unique identifiers, or web links for publicly available datasets
- A description of any restrictions on data availability
- For clinical datasets or third party data, please ensure that the statement adheres to our policy

The Cancer Therapeutics Response Portal (portals.broadinstitute.org/ctrp/) compound sensitivity dataset, a data matrix containing the normalized AUC values of each compound in each cell line, was downloaded from [https://ocg.cancer.gov/programs/ctd2/data-portal] [1-3]. Genome binding/occupancy profiling of SOX13 by high throughput sequencing have been deposited in Gene Expression Omnibus data base (GEO) under accession code GSE247870 [https://www.ncbi.nlm.nih.gov/geo/query/acc.cgi?acc=GSE247870]. The data of RNA-seq of ferroptosis-resistant GC cells generated in this study have been deposited in GEO under accession code GSE262114 [https://www.ncbi.nlm.nih.gov/geo/query/acc.cgi?acc=GSE262114]. The data of RNA-seq of GC cells transfected with shRNA-SOX13 or shRNA-NC have been deposited in GEO under accession code GSE211072 [https://www.ncbi.nlm.nih.gov/geo/query/acc.cgi?acc=GSE211072]. The data of untargeted metabolites of GC cells transfected with shRNA-SOX13 or shRNA-NC have been deposited in Metabolomics Workbench under accession code ST003134 [http://dx.doi.org/10.21228/M82431]. The remaining data are available within the Article, Supplementary Information or Source Data file.

1.Rees MG, Seashore-Ludlow B, Cheah JH, Adams DJ, Price EV, Gill S, Javaid S, Coletti ME, Jones VL, Bodycombe NE, Soule CK, Alexander B, Li A, Montgomery P, Kotz JD, Hon CS, Munoz B, Liefeld T, Dančík V, Haber DA, Clish CB, Bittker JA, Palmer M, Wagner BK, Clemons PA, Shamji AF, Schreiber SL. Correlating chemical sensitivity and basal gene expression reveals mechanism of action. Nat Chem Biol. 2016;12(2):109-16.

2. Seashore-Ludlow B, Rees MG, Cheah JH, Cokol M, Price EV, Coletti ME, Jones V, Bodycombe NE, Soule CK, Gould J, Alexander B, Li A, Montgomery P, Wawer MJ, Kuru N, Kotz JD, Hon CS, Munoz B, Liefeld T, Dančík V, Bittker JA, Palmer M, Bradner JE, Shamji AF, Clemons PA, Schreiber SL. Harnessing Connectivity in a Large-Scale Small-Molecule Sensitivity Dataset. Cancer Discov. 2015;5(11):1210-23.

3. Basu A, Bodycombe NE, Cheah JH, Price EV, Liu K, Schaefer GI, Ebright RY, Stewart ML, Ito D, Wang S, Bracha AL, Liefeld T, Wawer M, Gilbert JC, Wilson AJ, Stransky N, Kryukov GV, Dancik V, Barretina J, Garraway LA, Hon CS, Munoz B, Bittker JA, Stockwell BR, Khabele D, Stern AM, Clemons PA, Shamji AF, Schreiber SL. An interactive resource to identify cancer genetic and lineage dependencies targeted by small molecules. Cell. 2013;154(5):1151-1161.

Research involving human participants, their data, or biological material

Policy information about studies with human participants or human data. See also policy information about sex, gender (identity/presentation), and sexual orientation and race, ethnicity and racism.

Reporting on sex and gender	No selection bias was present.
Reporting on race, ethnicity, or other socially relevant groupings	Co-variate-relevant population characteristics of human research participants, e.g. age, gender, tumor size, lymph node metastasis, depth of invasion, histology, perineural Invasion, lymphovascular invasion, tumor SOX13/SCAF1 expression, tumor Regression Grade.
Population characteristics	Research specimen were blinded to clinical information related to this patient.
Recruitment	This study enrolled two independent cohorts consisting of 161 GC patients. In cohort 1, 109 fresh GC tissue pairs were collected from patients who underwent gastrectomy in Shanghai Cancer Center (Fudan University) between 2008 and 2012. In cohort 2, 52 endoscopic biopsy GC tissue pairs were obtained prior to cisplatin-based neoadjuvant chemotherapy in Yijishan Hospital (Wannan Medical College) between March 2019 and November 2020. The samples were collected and no intentional self-selection bias was present.
Ethics oversight	The study protocol was sanctioned by the local ethics committee of Yijishan Hospital Wannan Medical College and Fudan University Shanghai Cancer Center.

Note that full information on the approval of the study protocol must also be provided in the manuscript.

Field-specific reporting

Please select the one below that is the best fit for your research. If you are not sure, read the appropriate sections before making your selection.

☒ Life sciences ☐ Behavioural & social sciences ☐ Ecological, evolutionary & environmental sciences

For a reference copy of the document with all sections, see nature.com/documents/nr-reporting-summary-flat.pdf

Life sciences study design

All studies must disclose on these points even when the disclosure is negative.

Sample size	No sample-size calculation was performed, and the sample size are sufficient. 161 gastric cancer samples were used in this study. And at least 4 mice were in each group.
Data exclusions	Patients that have lost to follow up were excluded. The exclusion criteria were pre-established.
Replication	All findings were productive. three or four biologically independent repeats were performed to verify the reproducibility of the experiments.

	Sample sizes were large enough to ensure appropriate representation of the population behavior.
Randomization	Randomization was performed at the beginning of the xenograft experiments. All mice received control and experimental cells in each flank, controlling for mice to mice variation. No other experiments require randomization.
Blinding	The investigators were blinded to group allocation during data collection and analysis.

Reporting for specific materials, systems and methods

We require information from authors about some types of materials, experimental systems and methods used in many studies. Here, indicate whether each material, system or method listed is relevant to your study. If you are not sure if a list item applies to your research, read the appropriate section before selecting a response.

Materials & experimental systems

n/a	Involved in the study
☐	☒ Antibodies
☐	☒ Eukaryotic cell lines
☒	☐ Palaeontology and archaeology
☐	☒ Animals and other organisms
☒	☐ Clinical data
☒	☐ Dual use research of concern
☒	☐ Plants

Methods

n/a	Involved in the study
☐	☒ ChIP-seq
☐	☒ Flow cytometry
☒	☐ MRI-based neuroimaging

Antibodies

Antibodies used	SOX13 Rabbit polyclonal proteintech (18902-1-AP); COX7A2L/SCAF1 Rabbit Polyclonal proteintech (11416-1-AP); NADK Rabbit polyclonal Abcam (ab233261); NADK2 Rabbit monoclonal Abcam (ab181028); ACSL4 Rabbit monoclonal Abcam (ab155282);SLC7A11 Rabbit monoclonal Abcam (ab175186); GPX4 Rabbit monoclonal Abcam (ab125066); E-cadherin Mouse monoclonal Abcam (ab1416); Vimentin Rabbit monoclonal Abcam (ab92547); SDHA Mouse monoclonal Abcam (ab14715); MTCO1 Mouse monoclonal Abcam (ab14705); NDUFS2 Mouse monoclonal Abcam (ab110249); UQCRC2 Mouse monoclonal Abcam (ab14745); Trim25 Rabbit monoclonal Abcam (ab167154); FBXO28 Rabbit polyclonal Abcam (ab154068); TRAF2 Rabbit monoclonal Abcam (ab126758); β-Actin Rabbit polyclonal Abcam (ab8227)
Validation	SOX13 https://www.ptgcn.com/products/SOX13-Antibody-18902-1-AP.htm; COX7A2L/SCAF1 https://www.ptgcn.com/products/COX7A2L-Antibody-11416-1-AP.htm; NADK https://www.abcam.cn/nadk-antibody-ab233261.html; NADK2 https://www.abcam.cn/nadk2-antibody-epr13170-ab181028.html; ACSL4 https://www.abcam.cn/facl4-antibody-epr8640-ab155282.html; SLC7A11 https://www.abcam.cn/xct-antibody-epr82902-ab175186.html; GPX4 https://www.abcam.cn/glutathione-peroxidase-4-antibody-epncir144-ab125066.html; Vimentin https://www.abcam.cn/vimentin-antibody-epr3776-cytoskeleton-marker-ab92547.html;SDHA https://www.abcam.cn/sdha-antibody-2e3gc12fb2ae2-ab14715.html; MTCO1 https://www.abcam.cn/mtco1-antibody-1d6e1a8-ab14705.html; NDUFS2 https://www.abcam.cn/ndufs2-antibody-7a12be5ad5-ab110249.html; UQCRC2 https://www.abcam.cn/uqcrc2-antibody-13g12af12bb11-ab14745.html;Trim25 https://www.abcam.cn/trim25efp-antibody-epr7315-ab167154.html; FBXO28 https://www.abcam.cn/fbxo28-antibody-ab154068.html; TRAF2 https://www.abcam.cn/traf2-antibody-epr6048-ab126758.html; β-Actin https://www.abcam.cn/beta-actin-antibody-ab8227.html

Eukaryotic cell lines

Policy information about cell lines and Sex and Gender in Research

Cell line source(s)	Human GC cell lines were purchased from the American Type Culture Collection (ATCC). The mouse GC cell line (YTN16) was a gift from the laboratory of Jun Yu (The Chinese University of HongKong, HongKong)
Authentication	Short tandem repeat profiling was performed for authentication.
Mycoplasma contamination	All cell lines tested negative for mycoplasma.
Commonly misidentified lines (See ICLAC register)	No commonly misidentified cell lines were used in this study.

Animals and other research organisms

Policy information about studies involving animals; ARRIVE guidelines recommended for reporting animal research, and Sex and Gender in Research

Laboratory animals	BALB/c nu/nu athymic and C57BL/6 mice were 5–6 weeks old.
Wild animals	The study did not involve wild animals.

Reporting on sex	The findings apply to only female mice.
Field-collected samples	No field-collected samples were used in this study.
Ethics oversight	The animal experiments were carried out in strict accordance with protocols reviewed and approved by the Animal Care and Use Committee of Wannan Medical College.

Note that full information on the approval of the study protocol must also be provided in the manuscript.

Plants

Seed stocks	*Report on the source of all seed stocks or other plant material used. If applicable, state the seed stock centre and catalogue number. If plant specimens were collected from the field, describe the collection location, date and sampling procedures.*
Novel plant genotypes	*Describe the methods by which all novel plant genotypes were produced. This includes those generated by transgenic approaches, gene editing, chemical/radiation-based mutagenesis and hybridization. For transgenic lines, describe the transformation method, the number of independent lines analyzed and the generation upon which experiments were performed. For gene-edited lines, describe the editor used, the endogenous sequence targeted for editing, the targeting guide RNA sequence (if applicable) and how the editor was applied.*
Authentication	*Describe any authentication procedures for each seed stock used or novel genotype generated. Describe any experiments used to assess the effect of a mutation and, where applicable, how potential secondary effects (e.g. second site T-DNA insertions, mosiacism, off-target gene editing) were examined.*

ChIP-seq

Data deposition

☒ Confirm that both raw and final processed data have been deposited in a public database such as GEO.

☐ Confirm that you have deposited or provided access to graph files (e.g. BED files) for the called peaks.

Data access links<br>*May remain private before publication.*	GSE247870
Files in database submission	SNH-668-input_sequence_R1_fastqc; SNH-668-input_sequence_R2_fastqc; SNH-668-IP_sequence_R1_fastqc; SNH-668-IP_sequence_R2_fastqc; Peak_Annotation; Peak_Promoter_Annotation
Genome browser session<br>(e.g. UCSC)	*Provide a link to an anonymized genome browser session for "Initial submission" and "Revised version" documents only, to enable peer review. Write "no longer applicable" for "Final submission" documents.*

Methodology

Replicates	one replicate for input, one replicate for IP
Sequencing depth	ChIP-seq libraries were prepared using the KAPA HTP Library Preparation Kit complemented with NEXTflex DNA Barcodes from Bioo Scientific. 10 ng of DNA was used as starting material for input and ip samples. Libraries were amplified using 13 cycles on the thermocycler. Post amplification libraries were size selected at 250-450bp in length using Agencourt AMPure XP beads from Beckman Coulter. Libraries were validated using the Agilent High Sensitivity DNA Kit.
Antibodies	SOX13 Rabbit polyclonal proteintech (18902-1-AP)
Peak calling parameters	Peaks were called uing MACS v2.1.0 with the significance cut-off q-value <=0.01. bigWig files were generated using the genomicRanges R package. Score represents the normalized coverage of DNA fragments at a given genomic coordinate. narrowPeak files were generated using MACS v2 with default settings.
Data quality	21082 peaks at FDR 5% and above 5-fold enrichment
Software	MACS v2.1.0

Flow Cytometry

Plots

Confirm that:

☒ The axis labels state the marker and fluorochrome used (e.g. CD4-FITC).

☒ The axis scales are clearly visible. Include numbers along axes only for bottom left plot of group (a 'group' is an analysis of identical markers).

☒ All plots are contour plots with outliers or pseudocolor plots.

☒ A numerical value for number of cells or percentage (with statistics) is provided.

Methodology

Sample preparation	1.Sample preparation of patient peripheral blood cells:<br>1). Peripheral blood from patients with gastric cancer was collected;<br>2). Thoroughly mix 2mL fresh anticoagulant venous blood with 2mL 1xPBS buffer solution;<br>3). A 15 mL centrifuge tube was taken and 2 mL of lymphocyte isolation solution (Ficoll-Paque Plus) was added;<br>4). The mixture of 4 mL blood and PBS was slowly injected into the 2mL Ficol-Paque Plus liquid level along the tube wall using a pipetting device, paying attention to maintaining the stability of the stratified interface. The final peripheral blood, 1×PBS,Ficoll volume ratio was about 1:1:1;<br>5). Centrifuge with a horizontal rotor 400g for 30 min;<br>6). Collect the intermediate cell layer;<br>7). Wash twice with 1×PBS;<br>8). Flow cytometry antibody was added for staining.<br><br>2. Cell sample preparation:<br>Trypsin digested the cells and was collected, washed with PBS, and incubated with antibodies.
Instrument	CytoFLEX-FC500 MPL
Software	Collection software: CytExpert+2.0; Analysis software: FlowJo_v10.6.2 and Modfit 5.0
Cell population abundance	The abundance of target cell population in FSC and SSC channels reached more than 90%, and the rest were cell fragments.<br>The cell abundance of peripheral blood mononuclear cells was observed by Wright staining under a microscope.<br>Gastric cancer cells were purchased cell lines.
Gating strategy	1. Sample of patient peripheral blood cells:<br>1). Cells in normal group were taken as blank group and delineated by FSC-A and SSC-A channels;<br>2). Cell fluorescence intensity in blank group was negative group. If the fluorescence intensity is greater than blank group, it is positive group (The positive group was defined as the fluorescence intensity of CD4 and CD8 greater than 103 in PC7-A channel; The PE-A channel was used to detect INFγ. When the fluorescence intensity is greater than 103, it is positive group).<br><br>2. Cell sample:<br>1). Cells in normal group were taken as blank group and delineated by FSC-A and SSC-A channels;<br>2). The cell fluorescence intensity of the normal group was taken as the negative control group (fluorescence intensity was 10000-100000).<br>3). The fluorescence intensity of PE-A channel in the experimental group was 106-107.<br>4). The fluorescence intensity of CD45 positive cell group was 104 and that of CD45 negative cell group was 105.<br>5). When PI and ANNEXIN channels were used to detect apoptosis, the fluorescence intensity was higher than 105.<br>6). In the detection of C11, the positive peak was defined as the cell fluorescence intensity was greater than 105.

☒ Tick this box to confirm that a figure exemplifying the gating strategy is provided in the Supplementary Information.
